# Fourier-synthesis approach for static charge-density reconstruction from theoretical structure factors of CaB_6_

**DOI:** 10.1107/S2053273323002644

**Published:** 2023-05-05

**Authors:** Carina Bergner, Yuri Grin, Frank Richard Wagner

**Affiliations:** a Chemical Metals Science, Max-Planck-Institut für Chemische Physik fester Stoffe, Nöthnitzer Strasse 40, Dresden, Saxony 01187, Germany; University of Warsaw, Poland

**Keywords:** electron density, Fourier transformation, Fourier synthesis, hexaborides, Laplacian

## Abstract

In a pilot study, electron-density (ED) and ED Laplacian distributions were reconstructed for the challenging case of CaB_6_ (Pearson symbol *cP*7) with conceptually fractional B—B bonds from quantum-chemically calculated structure-factor sets with resolutions 0.5 Å^–1^ ≤ [sin(θ)/λ]_max_ ≤ 5.0 Å^–1^ by means of Fourier-synthesis techniques. Convergence of norm deviations of the distributions obtained with respect to the reference ones was obtained in the valence region of the unit cell. The QTAIM (quantum theory of atoms in molecules) atomic charges, and the ED and ED Laplacian values at the characteristic critical points of the Fourier-synthesized distributions have been analysed for each resolution and found to display a convergent behaviour with increasing resolution. The presented *method*(*exponent*) (ME) type of Fourier-synthesis approach can qualitatively reconstruct all characteristic chemical bonding features of the ED from valence-electron structure-factor sets with resolutions of about 1.2 Å^–1^ and beyond, and from all-electron structure-factor sets with resolutions of about 2.0 Å^–1^ and beyond. Application of the ME type of Fourier-synthesis approach for reconstruction of ED and ED Laplacian distributions at experimental resolution is proposed to complement the usual extrapolation to infinite resolution in Hansen–Coppens multipole model derived static ED distributions.

## Abstract 2

A novel type of Fourier-synthesis approach is reported for determining electron-density distributions and their Laplacians from static structure factors of CaB_6_. The approach relies on mathematical weighting functions to yield a data set, reproducing all characteristic chemical bonding features of the original quantum-chemically calculated distributions.

## 1. Introduction

As a consequence of the Hohenberg–Kohn theorems (Hohenberg & Kohn, 1964 ▸), the electron density (ED) of a chemical system is known to constitute its most fundamental observable property. Historically, in early X-ray diffraction experiments, only the locations of the atoms were identified from the dominating local maxima of the ED, which can be successfully modelled in the independent atom model (IAM), *i.e.* using a superposition of free atom form factors to model the structure factor for each reflection. With the improvement of experimental setups, the reconstruction of ED distributions from diffraction experiments came into reach, and methodological developments were devised for reliable extraction of this information from the observed diffraction intensities of elastic X-ray scattering experiments. Two fundamentally different strategies were followed for this purpose (Waser & Schomaker, 1953 ▸). One possibility consists of setting up a position-space model for the ED distribution and fitting it to the experimentally observed intensities. This was realized by replacing the superposition of spherically symmetric ED distributions in the IAM by a multipolar expansion, with the multipoles at each atomic site being consistent with the corresponding site symmetry. The most prominent of these methods used nowadays is the Hansen–Coppens (HC) multipole model (Hansen & Coppens, 1978 ▸). On the other hand, it was recognized early on that, in principle, the ED distribution can be reconstructed by ‘Fourier inversion’ of structure factors (Bragg & West, 1930 ▸). If this procedure is terminated before the remaining members of the Fourier series display negligibly small structure-factor amplitudes, series truncation artefacts like spurious local ED maxima and minima can occur, which adversely affect chemical interpretation. In the following, the process of Fourier inversion using a limited number of structure factors eventually multiplied by a weighting function will be called Fourier synthesis. It has long been known that multiplication of the static structure factors by the Debye–Waller factor helps to avoid series termination artefacts (Bragg & West, 1930 ▸). However, this turns the static ED distribution into a physically different, temperature-dependent dynamically smeared one. In pioneering experimental studies on Fourier synthesis of ED distributions in prototype ionic, polar covalent and covalent bonding situations in NaCl, MgO and diamond, the introduction of artificially enhanced temperature factors was found necessary in vibrationally hard compounds in order to eliminate series truncation artefacts within experimental resolution (Brill *et al.*, 1939 ▸, 1948 ▸).

Avoiding artificial temperature factors, a recent study reported on Fourier-synthesized dynamically smeared all-ED (all-electron density) distributions for two amino acids free from series truncation effects (Mondal *et al.*, 2012 ▸). They were calculated from static structure factors and atomic displacement parameters obtained by HC multipole model fits of temperature-dependent X-ray diffraction data with resolutions [sin(θ)/λ]_max_ ≤ 1.2 Å^–1^. Good convergence of the synthesized dynamical all-ED and all-ED Laplacian distributions was shown to require usage of extrapolated structure factors from the fitted multipole model up to cut-offs at [sin(θ)/λ]_max_ ≥ 6 Å^–1^. Note that this extrapolation to high resolution was necessary because of a combination of very strict numerical convergence conditions with comparably small physical vibrational smearing working against series termination effects. In order to perform Fourier synthesis using only HC dynamical structure factors within experimental resolution, a suitable mathematical weighting function may be invoked.

Notably, even the practice in standard HC studies on experimental EDs to calculate the static ED distributions from fitted atomic multipoles in real space (avoiding Fourier synthesis) corresponds to an implicit extrapolation of the HC model structure factors to infinite resolution (Coppens & Stevens, 1977 ▸). An alternative route more consistent with the experimental conditions would be the Fourier synthesis of the ED using only those static structure factors of the HC model that correspond to the experiment resolution. To the best of our knowledge, this has not yet been done.

In the present methodological study, the focus lies on mathematical weighting functions in order to keep the physical interpretation of the original data set, *i.e.* Fourier synthesis using static ED structure factors and mathematical weighting functions still yields (smoothed) static ED distributions. The effectiveness of a number of mathematical weighting functions on convergence of Fourier synthesis of static valence (val)-EDs and all-EDs and their Laplacians is investigated. One reason for selection of static ED Fourier synthesis is that there is a theoretical foundation for a QTAIM (quantum theory of atoms in molecules) (Bader, 1990 ▸) topological study for static EDs. Moreover, the focus on static ED synthesis represents a more challenging task compared with the dynamic one, because series termination effects and artefacts are more prominent there. By converging the Fourier synthesis of static EDs already at experimental resolution with suitable mathematical weighting functions, the extrapolation of structure factors or usage of artificially enhanced Debye–Waller factors can be avoided. The same technique may also work for Fourier synthesis of dynamic EDs, thus avoiding extrapolation of experimental data sets (see above).

Finally, the HC model fitting is not free from difficulties and biases (Michael & Koritsanszky, 2017 ▸), such that the fitted structure factors {*F*_HC_} may be different from the initially given ones {*F*_0_}. It is conceivable that, in certain cases, significantly different ED or ED Laplacian distributions may be obtained compared with those obtained from Fourier synthesis using the initial structure-factor set {*F*_0_}.

On the methodological side, static EDs in general are available only from a model. In the present case, a quantum-chemical DFT (density functional theory) calculation on a crystalline system was employed, and the ED Fourier coefficients (structure factors) can be obtained up to arbitrary resolution. The static ED and its Laplacian calculated from the model correspond to the reference distributions in the subsequent investigations, and the various Fourier-synthesized distributions were evaluated with respect to these references not only at critical points, but also in the whole unit cell and parts of it using norm deviations.

**Figure 1:**
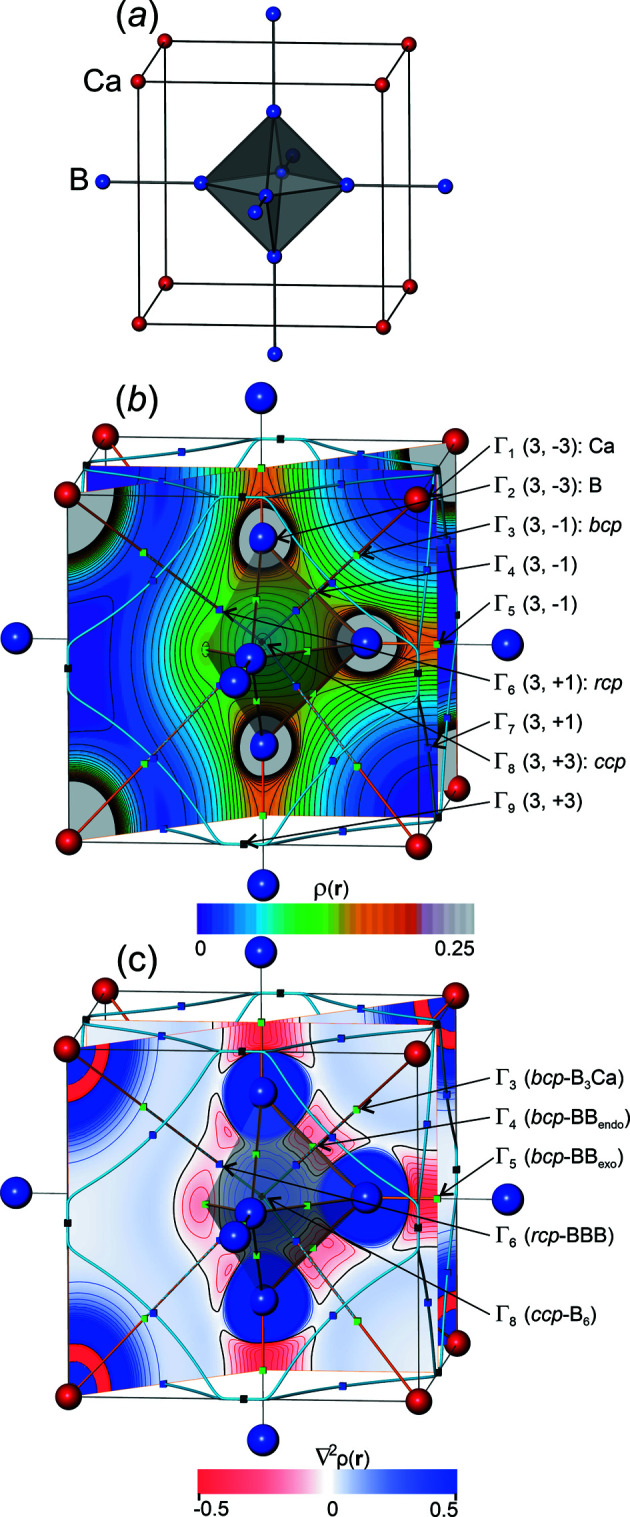
(*a*) Crystal structure of CaB_6_ (space group 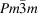 ); unit-cell box is shown by black lines, B—B bonds by grey lines. (*b*) Reference ED and (*c*) reference ED Laplacian distributions of CaB_6_ in the (200) and (110) planes with molecular graph; Γ*_i_* (*rank*, *signature*) enumerates and classifies the crystallographically independent ED critical points; ED isoline steps at 0.01 e^−^ bohr^−3^, ED Laplacian isoline steps of 0.05 e^−^ bohr^−5^.

As an example test case, the non-molecular crystal structure of CaB_6_ has been chosen (employed experimental structure data in Table 1 ▸). The proper reconstruction of its ED features, which are related to a rather evolved chemical bonding scenario, is considered to be methodologically more challenging than for a molecular crystal of a classical organic molecule like, *e.g.*, glycine or urea. The cubic structure [space group 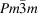 , Fig. 1 ▸(*a*)] of binary hexaborides *M*B_6_ (*M* = Na, K, Rb, Ca, Sr, Ba, Sc, Y, La) is based on a CsCl-like arrangement of metal atoms and barycentres of sixfold interconnected B_6_ octahedra. The octahedral B_6_ clusters display six short inter-octahedral (*exohedral*) and 12 longer intra-octahedral (*endohedral*) B–B contacts with substantially different bonding character. Conceptually, the exohedral contacts represent single two-centre (2c) bonds, while the endohedral ones are described by a mixture of 2c and three-centre (3c) bonding character (Longuet-Higgins & Roberts, 1954 ▸). A recent theoretical study focuses on the description of chemical bonding for binary hexaboride compounds using position-space bonding indicators, namely the ED distribution ρ(**r**), the ED Laplacian ∇^2^ρ(**r**), as well as the pair density based electron localizability indicator distribution (ELI-D), and 2c and 3c delocalization indices (DIs) between atomic regions (Börrnert *et al.*, 2013 ▸) (the first author of the present paper, Dr Carina Bergner, was previously known as Dr Carina Börrnert). The resulting QTAIM effective charge Ca^1.52+^ is consistent with formal charge assignment Ca^2+^ obtained on the basis of ELIBON (ELI-D-based oxidation number). This is in accordance with the saturation of electronic demand of the boron framework composed of 6-connected *closo* B_6_^2−^ clusters by charge transfer of two electrons from Ca according to the Lipscomb–Wade rules (Lipscomb, 1979 ▸; Wade, 1971 ▸). Two- and three-centre DI analysis reveals a 2c–2-electron character of the exohedral B—B bonds, and – in agreement with ELI-D topology – a mixed 2c + 3c character of endohedral B—B bonding. Endohedral 2c DIs of about 0.60, and 3c ones of about 0.20 are found for electronically saturated hexaborides of the alkaline-earth metals. These values are related to the total amount of seven endohedral bonds expected according to Lipscomb–Wade rules (Börrnert *et al.*, 2013 ▸).

The pair density based bonding indicators ELI-D and DIs are still not accessible in purely experimental studies of all types of crystalline compounds. The very useful X-ray constrained wavefunction calculation method, which delivers this information at a semi-experimental level, is nowadays still technically limited to molecular crystals (Genoni & Jayatilaka, 2021 ▸). Using diffraction experiments, the ED distribution can be reliably reconstructed from structure factors. Certain specific features of chemical bonding in hexaborides can already be obtained from ED-based quantities, *e.g.* QTAIM atomic charges and charge-density concentrations [∇^2^ρ(**r**_c_) < 0] at bond and ring critical points **r**_c_. They fit the generally accepted bonding picture. In the quantum-chemical calculations on CaB_6_, comparably high ED is found within the B_6_ skeleton, *i.e.* edges and faces (Mebs *et al.*, 2011 ▸), and lower values in the octahedron centre and interstitial regions [Fig. 1 ▸(*b*)]. The topological features of the ED distribution, namely the critical points **r**_c_ obeying ∇ρ(**r**_c_) = 0, have been determined within the unit cell and characterized by their curvatures (Börrnert *et al.*, 2013 ▸; Börrnert, 2013 ▸). The set of all critical points of types (*rank*, *signature*) within the unit cell [Fig. 1 ▸(*b*)] satisfies the Poincaré–Hopf relationship (Zou & Bader, 1994 ▸). The distribution of the ED Laplacian [Fig. 1 ▸(*c*)] contains additional information about particular chemical bonding features in CaB_6_. Values of ∇^2^ρ < 0 at the bond critical points (b.c.p.) Γ_4_ [*d*(B—B)_endo_ = 1.751 Å] and Γ_5_ [*d*(B—B)_exo_ = 1.676 Å] between the boron atoms indicate a charge concentration which is characteristic for covalent bonding within the boron framework. Apart from the Ca-atom locations, the inter-octahedral region of the CaB_6_ structure is characterized by low values of ρ. A b.c.p. Γ_3_ is found between the octahedral face and the Ca atom [Fig. 1 ▸(*b*)]. The positive value of ∇^2^ρ at and close to this b.c.p. represents charge depletion and indicates a predominantly ionic interaction between the metal atoms and the boron framework. Negative values of ∇^2^ρ not only along the endohedral B—B bond path and b.c.p. Γ_4_, but also around the B_6_ clusters, and especially at the ring critical points (r.c.p.) Γ_6_ slightly above the octahedral triangular face midpoints (Bader & Legare, 1992 ▸), mark the whole endohedral boron skeleton (*i.e.* B—B bonds and B—B—B triangles) as a region of charge-density concentration (∇^2^ρ < 0). Charge-density depletion is found for the interior part of the octahedron, with the highest depletion found at the cage critical point (c.c.p.) Γ_8_ at the octahedron centre. Summarizing, it is evident that for CaB_6_ ED characteristics qualitatively feature certain results of pair density based bonding indicators important for chemical understanding of this type of compound.

The purpose of this work is to investigate, on the basis of the calculated ED and ED Laplacian distribution for CaB_6_ (structure parameters, Table 1 ▸) from a periodic DFT calculation, whether its decisive chemical bonding features can be extracted from a data set of static structure factors with a given resolution ½*H*_max_ = [sin(θ)/λ]_max_. Within this analysis, reconstructed distributions of ρ and ∇^2^ρ for CaB_6,_ obtained by different Fourier-synthesis methods, are compared with each other and with the original distributions obtained from DFT calculation at the experimental geometry. All structure factors were calculated from the DFT wavefunction (see Appendix *A* [App appa]). Comparison of the relevant chemical bonding features of the quantum-chemically calculated static reference ED with those obtained from a suitable Fourier synthesis at experimentally accessible resolution [sin(θ)/λ]_max_ can give an answer to the question of whether that resolution is sufficient to yield the chemical bonding information content of the original distribution. This means a suitable Fourier-synthesis procedure may be a valuable tool to evaluate the ideally extractable information content of the original ED from a certain measured or measurable data set with limited resolution.

## 2. Methods

### 2.1. ED and ED Laplacian distributions from theoretical structure factors by the *method*(*exponent*) type of Fourier-synthesis approach

The ED ρ(**r** *)* of a crystalline system is physically observable. Diffraction measurements on crystalline samples yield diffraction intensities *I*(**H**) at reciprocal-lattice positions 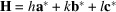 , which are related to the squared modulus of the product of the corresponding static structure factors *F*(**H**) and the Debye–Waller factor. These material- and structure-dependent quantities are obtained by fitting intensities of a coherent elastic scattering experiment, *e.g.* by means of the HC pseudo-atom model (Hansen & Coppens, 1978 ▸; Gillet & Koritsanszky, 2012 ▸).

The ED structure factors are defined by a Fourier integral of the corresponding ED distribution ρ(**r**) in the unit cell (u.c.), the static or the dynamically smeared one. For the purpose of a subsequent QTAIM analysis, we focus on static EDs and structure factors *F*(**H**) in the following,

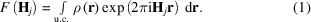

Mathematical inversion of this formula leads to the well known Fourier-series representation of the ED (Waser & Schomaker, 1953 ▸):

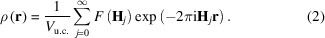

Exact reconstruction of the original ED via Fourier back-transformation is impossible in practice due to the necessity of infinite structure-factor sets; all procedures working with such incomplete back-transformations will be called Fourier synthesis in the following,

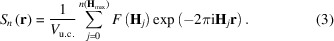

**Figure 2:**
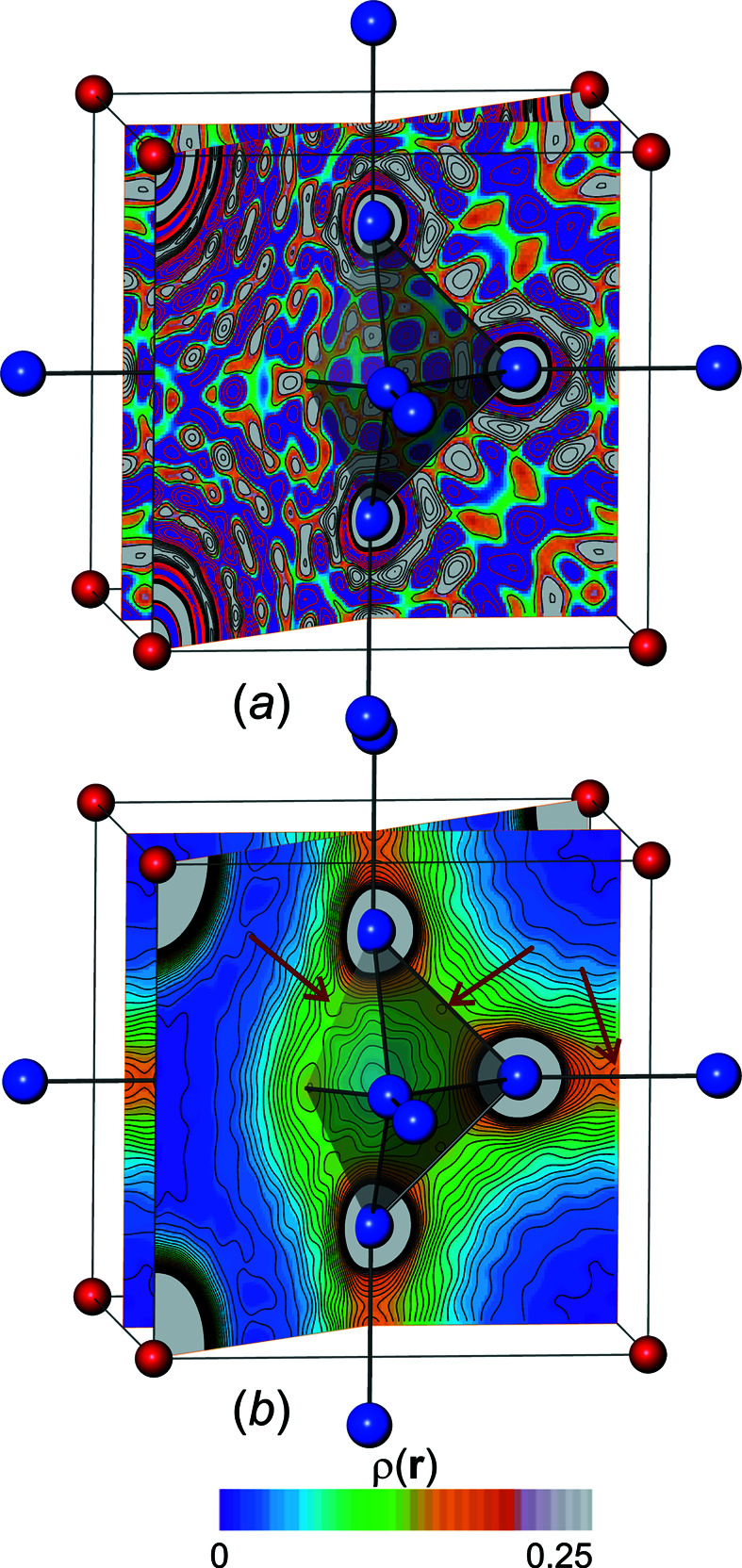
Raw Fourier synthesis [equation (3)] of the ED distribution for CaB_6_ at [sin(θ)/λ]_max_ = 1.5 Å^−1^ shown in the (200) and (110) planes. (*a*) Synthesized all-ED *S_n,_*_tot_(**r**) with spurious NNMs and large regions with negative values (red isolines). (*b*) All-ED 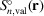 from val-ED synthesis *S_n,_*_val_(**r**) [equation (19)] with spurious NNMs, the most prominent ones shown by red arrows; black isoline steps of 0.0078 e^−^ bohr^−3^.

The incompleteness of Fourier series *S_n_* (**r**) often leads to negative ED values, spurious non-nuclear maxima (NNMs) and other errors, *e.g.* shifted nuclear ED maxima, broadened nuclear ED ‘peaks’ and ED ripples (Altomare *et al.*, 2008 ▸), seen in the raw Fourier-synthesized [equation (3)] EDs [Fig. 2 ▸(*a*)]; all of them are typically classified as series termination errors. In the present Fourier-synthesis approach for ED and ED Laplacian reconstruction for QTAIM analysis, we tend to distinguish between series truncation artefacts (negative ED values and NNMs not present in the reference ED) and so-called (normal) series termination errors. The latter correspond to all other deviation types of a limited-resolution Fourier synthesis from the mathematically exactly [at infinite resolution, equation (2)] back-transformed ED. An enumeration of deviation types is typically dependent on context, such that for the present QTAIM type of ED analysis, additional series termination errors were even more relevant than those mentioned before, namely shifts of positions of critical points, deviations of ED and ED Laplacian values at critical points, and ED Laplacian ripples. The distinction between artefacts and errors reflects the evaluation strategy adopted in the present approach. Fourier-synthesis results with ED features classified as series termination artefacts are considered of minor quality and are usually dropped. In contrast, Fourier-synthesis results displaying (normal) series termination errors (in the present definition) are principally accepted while working systematically to decrease them (see below). The classification as series termination artefact is clearly possible for the negative ED values, which are not allowed for real EDs; the situation is more delicate for NNM occurrence. NNMs are principally allowed features for real ED distributions, though rather seldom occur. Their occurrence during Fourier synthesis of an unknown original ED should be carefully checked in order to either establish their reality or identify them as artefacts. This would be related to potential future applications as a stand-alone method and not for the present study, where they are clearly identified as artefacts, because the reference DFT ED from which the ED structure factors have been obtained does not display NNMs.

*Weighting-function methods*. 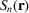 is mathematically defined by folding the true distribution of ρ(**r**) with the Dirichlet kernel *D_n_* (**r**). The latter is a cosine series oscillating in sign and value around *D_n_* (**r**) = 0 (Pepinsky, 1952 ▸). This is the reason why Fourier synthesis with this kernel (‘raw Fourier synthesis’) may even yield regions in space where *S_n_* (**r**) < 0. ED distributions obtained from partial sums *S_n_* (**r**) are characterized by Euclidean norm deviations 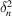 , which monotonically decay with increasing number *n* of partial sum elements ( 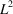 norm) (Carleson, 1966 ▸):

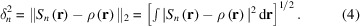

For a given distribution *S_n_* (**r**), a reduction of the truncation errors can be achieved by modifying the Dirichlet kernel, evoking a gradual down-weighting of partial sum elements, *i.e.* structure factors, with increasing length 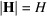 of the scattering vector. This automatically occurs already by multiplication of the static structure factor with the Debye–Waller factor, which decays as exp(–¼*B*_iso_ *H* ^2^) (Coppens, 1997 ▸). It results from folding the static ED distribution with the thermal motion of the atoms. In order to smooth truncation errors, early works on ED reconstruction either suggested the introduction of an artificial temperature factor (Waser & Schomaker, 1953 ▸; Pepinsky, 1952 ▸) or extrapolation of the measured data using artificial series members (van Reijen, 1942 ▸). Acceleration of the convergence of the series expansion by substituting the strongly oscillating Dirichlet kernel by integral kernels with invariably positive terms offers a purely mathematical method to avoid series truncation artefacts and reduce certain errors without using empirical functions (Pepinsky, 1952 ▸). The most prominent of those kernels is the Fejer kernel, which is obtained by Cesaro summation, *i.e.* calculating the arithmetic average *C*_*nmax*_(**r**) of partial sums *S_n_* (**r**) defined in equation (3):

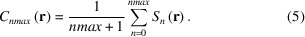

This leads to a maximum weight of 1.0 for the lowest structure factor *F*(000) = *F*(**H** *_j_*_=0_), and a monotonic decrease of the weights σ_C_(**H** *_j_*) of the remaining *nmax* structure factors *F*(**H** *_j_*) according to 1 − *n*/(*nmax*+1) along the sequence *n* = 0, 1,…*nmax*. For this reason, the non-negative Fejer kernel acts as a low-pass filter on the structure factors, which leads to uniform convergence of a Fourier series.

As can be seen from the 1D index *n*, the Fejer method historically is a 1D method, where the Cesaro summation of partial sums of Fourier coefficients finally leads to a simple multiplicative factor σ_C_(**H** *_j_*) for each Fourier coefficient *F*(**H** *_j_*) in the Fourier-synthesis formula [equation (6)]:

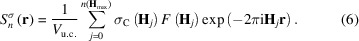

For a given resolution [sin(θ)/λ]_max_ = ½*H*_max_ and a corresponding number *n*(*H*_max_)+1 of Fourier coefficients to be included, the size for each σ_C_(**H** *_j_*) factor varies in the range ]0, 1] and is related to the position of the Fourier coefficient in the sequence of coefficients *j* = 0…*n*(**H**_max_), according to 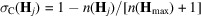 , where *j = n*(**H**_max_) denotes the final coefficient to be included with non-zero weight. Application of this method to structure-factor sets {*F*(**H** *_j_*)} organized by three indices *h_j_*, *k_j_*, *l_j_* is a non-unique extension. Such a 3D variant for ED reconstruction has been proposed and applied by Pepinsky (1952 ▸). It corresponds to σ_C_(**H** *_j_*) factors composed of a product of the three 1D terms related to *h_j_*, *k_j_* and *l_j_*:

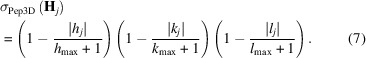

All symmetry-related structure factors [**H** *_j_*]_sym_ belonging to the same reflection class obtain the same weighting factor, which is a common feature of all weighting schemes. As a special feature of all 3D weighting schemes presented herein, symmetry-independent structure factors with the same ½|**H** *_j_|* = sin(θ)/λ obtain different weighting factors. Note that this weighting method denoted Pep3D in the following has some intrinsic disadvantage. It selects [equation (7)] a box-shaped region from the available structure factors *F_hkl_*, which introduces a certain directional anisotropy. Nevertheless, numerical evaluation of this method has been selected herein also for historical reasons.

Utilizing the degrees of freedom in the weighting schemes denoted as 1D in the following, an ordered string (1D) of structure factors {*F*(**H** *_j_*)} is formed using the natural sequence of increasing *H*_*j*_ = |**H** *_j_* |. So, the ordered string looks like {[*H_j_*]_|**H**|_}_ord_. Not only will all symmetry-related structure factors be given the same weighting factor, but so will all reflections with the same *H_j_* in general. This is a necessary consequence of having a uniquely defined sequence of structure factors with increasing *H_j_* leading to monotonically decaying sigma factors with *H_j_*. In the Fejer spirit, we may now assign increasing position numbers *n_j_* to each class [*H_j_*]_|**H**|_. *F*(0, 0, 0) has to be assigned position number *n*_0_ = 0, as it must always obtain a weighting factor of 1. Three different ways to assign position numbers for the remaining list of structure-factor classes [*H_j_*]_|**H**|_ were investigated.

In the conceptually simplest case (at first sight), we may assign consecutive integer position number values *n_j_* = *j* = 1, 2,…, *N*_max_ to the sequence of *N*_max_ classes [*H_j_*]_|**H**|_. As an example, the first three classes [0,0,0] _|**H**|_, [1,0,0] _|**H**|_ and [1,1,0]_|**H**|_ in the ordered list obtain position numbers *n_j_* = 0, 1 and 2, respectively. This weighting method is denoted Fej_cnt in the following:

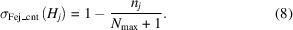




In the variant denoted Fej_pcl, we take into account the number of structure factors inside each [*H_j_*]_|**H**|_ class. The procedure uses the consecutive integer counting numbers *j* of each single structure factor in the complete ordered list. Each of the *N*_max_ classes [*H_j_*]_|**H**|_ obtains the position value of the arithmetic mean of the first and the last member’s position value, *n_j_* = (*j*_first_ + *j*_last_)/2. As an example, the first classes in the list [0,0,0] _|**H**|_, [1,0,0] _|**H**|_ and [1,1,0]_|**H**|_ obtain position numbers 0, 3.5 and 12.5, respectively. This procedure may be justified considering that an infinitesimal, *e.g.* orthorhombic, distortion would keep the number of structure factors, but split the [*H_j_*]_|**H**|_ classes and lead to additional position numbers compared with Fej_cnt. These are consistently taken into account in the Fej_pcl weighting method:

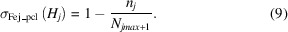




Having accepted position numbers for the [*H_j_*]_|**H**|_ classes being fractional and not necessarily increasing by 1, the introduction of Fej_stl represents only a small additional step. In this weighting scheme there is an infinite number of position number holes between the present ones, keeping the space for additional structure factors (reflections), *e.g.* in case of symmetry reductions that lead to vanishing of non-primitive lattice translations. This is effectuated by using the *H_j_* values themselves as ‘position numbers’. Moreover, this 1D scheme is a directly related alternative to the 3D Pepinsky one. Instead of using the product of three 1D terms, the *h*, *k*, *l* values are additively combined according to 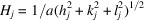 for the cubic structure [*H_j_* = 1/*d*_*hkl*_, *i.e.* the reciprocal interplane distance of lattice planes (*h_j_ k_j_ l_j_*)], with ‘*a*’ being the lattice parameter:

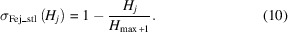




Since ½*H_j_* = sin(θ)/λ, weighting factor σ_Fej1D_ will linearly decrease with sin(θ)/λ. Note that all 1D schemes presented herein automatically avoid the problem of selecting structures *F_hkl_* in a box-shaped region like in the original Pep3D [equation (7)]. The region defined by *H*_max_ is always spherical in reciprocal space.

Based on Lanczos’s solution of smoothing the first derivative of a 1D Fourier expansion (Lanczos, 1956 ▸), Shchedrin & Simonov (1969 ▸) suggested an algorithm for the 3D-expanded ED Fourier-synthesis problem. Although the authors were not interested in the ED gradient itself, it was recognized that smoothing the ED gradient simultaneously smooths the contours of the ED itself. Therefore, the Lanczos factors can serve the same purpose as the Fejer factors, the smoothing of the ED to counteract series termination artefacts:

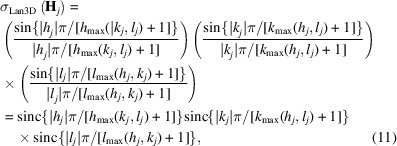

with

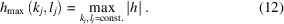

This weighting method, herein denoted Lan3D, has been implemented and employed in the following. Note that the special condition [equation (12)] was especially designed to avoid box-type selection of structure factors as occurs in the original Pep3D [equation (7)].

Interestingly, a similar weighting function has also been used for Fourier back-transformation of magnetic structure factors obtained from polarized neutron scattering experiments to calculate experimental magnetization density maps (Shull & Mook, 1966 ▸).

In analogy to method Fej_stl, a 1D Lanczos method has been set up, called Lan1D in the following. It is possibly identical to the method mentioned by Strel’tsov *et al.* (1985 ▸), but without the explicit formulas given there. Similar to the 1D Fejer methods, in the Lan1D weighting scheme all structure factors with the same *H_j_* obtain the same numerical weighting-factor value, while this is not so in the 3D schemes Pep3D and Lan3D:

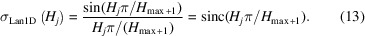

With these six weighting-function methods for compensation of series termination effects in the Fourier synthesis of the ED distribution at hand, the question remains as to how to achieve a smoothed ED Laplacian distribution?

*Weighting-function exponent*. While Fourier series in general are convergent, this is not necessarily valid for their derivatives. Caused by the additional factor 4π^2^ **H** *_j_* ^2^, which increases with increasing size (resolution) of the Fourier series faster than the static structure factors decay, the second derivatives of these Fourier series are divergent,

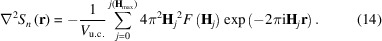

As already mentioned, the Lanczos scheme was initially set up to suppress oscillation artefacts in the first derivative of a truncated Fourier series. Clearly, these artefacts are already contained in the synthesized ED distribution itself, and a numerical differentiation of this distribution gives the same artefacts as the analytical one. Therefore, smoothing out series termination artefacts of the ED will simultaneously improve the ED gradient and Laplacian representation as well. The interconnection between the ED distribution, its gradients and its Laplacian distribution is directly employed in the QTAIM topological analysis of the ED distribution. The critical points are located where the ED gradient is zero, and they are characterized by the ED curvatures at this point. Therefore, the ED, the ED gradient and the ED Laplacian have to be synthesized using the same sigma factor σ^*p*^(**H** *_j_*). This simple conclusion also implies a further degree of freedom in the sigma factor’s usage. Since the initial sigma factor derived by Lanczos was for the first derivative, the Laplacian should have the squared sigma factor, both of which cannot be fulfilled simultaneously in the topological analysis of the ED. This not only means that one can use either exponent, 1 or 2, but that one can use any real number *p* ≥ 1.0 (and eventually even *p* > 0). Exploiting this degree of freedom has been found vital for the whole study. To the best of our knowledge, this has never been considered or formulated before in the framework of Fourier synthesis of EDs and ED Laplacians. As a strategy, for each Fourier-synthesis method presented herein, the lowest exponent *p* has been used, which yields a series of partial sums *S_n_* (**r**) without artefact NNMs at ½*H*_max_ > 1.1 Å^−1^. For the present study shown below, this was achieved with 1.0 ≤ *p* ≤ 3.0, with the actual value used depending on the method. The specification of the weighting function used in each case will be abbreviated in the form *method*(*p* value), *e.g.* Lan1D(1.75). Summarizing, for Fourier synthesis the following equations were always employed:

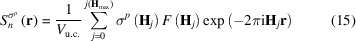




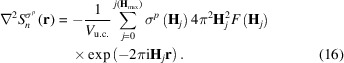

In the following, the σ^*p*^ superscript of the synthesized distributions 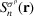 [equation (15)] and 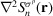 [equation (16)] indicating the present *method*(*exponent*) (ME) type of approach is omitted for brevity. The effect of the σ*^p^* factors with respect to the convergence of the ED and its Laplacian distribution with increasing resolution ½*H*_max_ is investigated using the example of CaB_6_.

Although the application of Fourier synthesis for ED reconstruction and even for its Laplacian has been reviewed (Tsirelson & Ozerov, 1996 ▸), so far, the approach itself (independent from experimental uncertainties) has never been systematically investigated with respect to convergence behaviour of QTAIM-related properties. Typically, only the final results obtained from the experimental [sin(θ)/λ]_max_ are reported, but the behaviour of the final values upon increasing resolution from lower resolutions up to this final one would be a further criterion to judge reliability of the final results. Moreover, as explained above, in a QTAIM study the Fourier syntheses of the ED and the ED Laplacian are conceptually restricted [equations (15), (16)] to employ the same smoothing factors σ*^p^* (**H** *_j_*). To the best of our knowledge, it has never been explicitly noted in the literature that this has actually been done.

*Valence-electron and all-electron Fourier synthesis*. A partitioning of all-electron density (all-ED) distributions into additive core-electron (core-ED) and valence-electron (val-ED) distributions has been found useful in chemistry, physics and crystallography, *i.e.* for the reference DFT all-ED and all-ED Laplacian, one can formulate

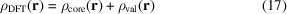

and

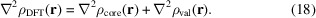




The core states of a compound, *e.g.* B(1*s* ^2^), Ca(1*s* ^2^, 2*s* ^2^, 2*p* ^6^, 3*s* ^2^, 3*p* ^6^) for CaB_6_, are assumed to be chemically inert, such that the core-ED can be reasonably approximated by the core-ED from free atoms, and the chemical focus typically lies on characteristic features of the val-ED specific for the respective compound. In the present study, Fourier syntheses of val- and all-EDs and their Laplacian distributions are investigated. They are computed from application of equations (15), (16) using val-ED and all-ED structure factors obtained for the reference DFT wavefunction from the program *Elk* (see Appendix *A* [App appa]). With the subsequent QTAIM analysis in mind, in the present study all-ED and all-ED Laplacians are always employed and evaluated with respect to the corresponding reference DFT all-electron distributions. All-ED and all-ED Laplacian distributions synthesized from all-ED structure factors are denoted *S_n_*_,tot_(**r**) and 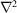 S*_n_*_,tot_(**r**), respectively, in the following. For synthesized val-ED distributions *S_n_*_,val_(**r**) and 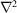 S*_n_*_,val_(**r**) only, an additional step is required to construct a special kind of all-ED distribution 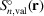 and 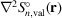 by adding the corresponding reference DFT core-ED distributions ρ_core_(**r**) and 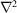 ρ_core_(**r**):

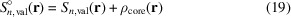




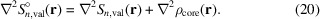




Calculated deviations (see the next section) of this kind of all-ED [equation (19)] and all-ED Laplacian [equation (20)] with respect to the corresponding reference DFT all-ED [equation (17)] and all-ED Laplacian [equation (18)] can be easily seen,

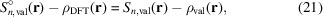




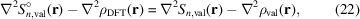

to solely measure the val-ED and val-ED Laplacian deviations with respect to the corresponding reference DFT val-ED and val-ED Laplacian distributions.

### 2.2. Statistical and topological evaluation of Fourier-synthesized EDs and ED Laplacians with respect to deviations from reference DFT-based distributions

For evaluation of obtained ED and ED Laplacian distributions *S_n_* (**r**) and 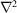 *S_n_* (**r**) determined with increasing resolutions ½*H*_max_ by various Fourier-synthesis *methods*(*exponents*), several statistical quality and convergence measures have been calculated. They are all based on the norms of the deviations of the obtained distributions from the reference distributions ρ_DFT_(**r**) and 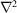 ρ_DFT_(**r**).

With the ED ρ(**r**) being a square-integrable function, the sequence of its Fourier-synthesized distributions *S_n_* (**r**) is expected to converge with the number *n* of structure factors in the Euclidean norm δ*L* ^2^ (Riesz–Fischer theorem, more precisely δ*L^m^*, with 2 ≤ *m* < ∞), *i.e.*


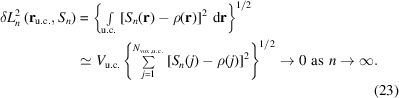

A different way to perform Fourier synthesis proceeds via Fejer summation, which can be written as a weighted Fourier sum (see above). This kind of summation is expected to show uniform convergence (Fejer’s theorem), and converges already in the δ*L* ^1^ norm,

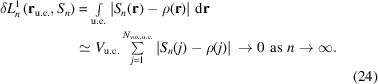




For the criterion of ‘uniform convergence’ to be fulfilled, the maximum norm δ*L* ^∞^ has to converge according to

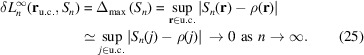

Note that uniform convergence always implies Euclidean and point-wise convergence, but not vice versa (Tveito & Winther, 2005 ▸). The maximum norm Δ_max_(*S_n_*) describes the maximal deviation between the original and the reconstructed ED in the unit cell. It is a very transparent measure of convergence, although, when calculated on a finite grid, it may be more prone to missing positions with higher deviations than the mean square convergence measure (seen in one case below). Note that the position **r** of maximal deviation may change with increasing resolution.

The actual computations were performed by discrete summations over an orthogonal equidistant real-space mesh of *N*_vox,u.c._ = 158 × 158 × 158 grid voxels *j* (mesh size = 0.02628 Å) covering the whole CaB_6_ unit cell. The all-electron [*F*_tot_(000) = 50 e^−^] and valence-electron [*F*_val_(000) = 20 e^−^] density structure factors were calculated from the DFT wavefunction of the full unit cell. The ED and ED Laplacian distributions obtained from Fourier synthesis were evaluated on the uniform grid in the full unit cell using the δ*L_n_* ^1^, δ*L_n_* ^2^ and δ*L_n_* ^∞^ norm deviations with respect to the reference DFT distributions ρ_DFT_(*j*) and 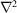 ρ_DFT_(*j*).

The δ*L_n_* ^1^ and δ*L_n_* ^2^ norm deviations per voxel of the reconstructed val-EDs *S*_*n*,val_(**r**) and their Laplacians 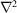 *S*_*n*,val_(**r**) (replace ‘ 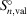 ’ by ‘ 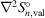 ’), and all-EDs *S*_*n*,tot_(**r**) and their Laplacians 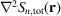 (replace ‘*S*_*n*,tot_’ by ‘ 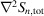 ’) with respect to the reference DFT all-ED ρ_DFT_(**r**) and its Laplacian have been calculated on the unit-cell grid positions **r**_u.c._:

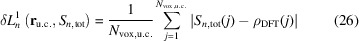




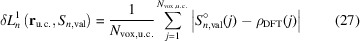




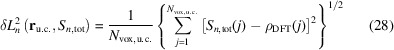




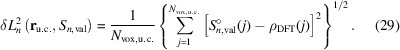

In the present study, the focus lies mainly on the critical point reconstruction between the atoms. Therefore, the statistical measures introduced have been computed additionally for the valence region between the atomic core regions. Summation over the voxels of the valence region (*N*_vox,val_ = 3327147) was performed, skipping the voxels inside the spherical regions around the atomic positions with radii of 2.495 and 0.75 bohr around Ca and B atoms, respectively, which are identified as core regions from ELF/ELI-D (ELF = electron localization function) atomic shell structure studies of free atoms (Kohout & Savin, 1996 ▸; Baranov, 2014 ▸):

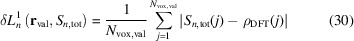




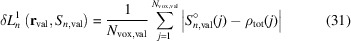




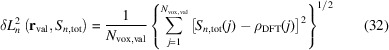




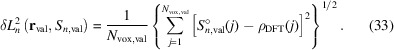

While the theorems on the convergence are valid for the full region of the unit cell (denoted **r**_u.c._), it is not clear how the statistical deviation measures behave in the valence region (denoted **r**_val_).

For all distributions calculated herein δ*L_n_* ^1^ and δ*L_n_* ^2^ norm deviations of the same distribution were found to obey the known relations δ*L_n_* ^1^ > δ*L_n_* ^2^ and δ*L_n_* ^1^ < *N*_vox_^1/2^ δ*L_n_* ^2^, as mathematically expected. Moreover, for all distributions calculated herein, it was also found that for each Fourier-synthesis function, the absolute deviations in the valence regions **r**_val_ were smaller than the corresponding ones in the full regions, δ*L_n_* ^1^(**r**_val_) < δ*L_n_* ^1^(**r**_u.c._) and δ*L_n_* ^2^(**r**_val_) < δ*L_n_* ^2^(**r**_u.c._), which is caused by the rather different maximal ED values in both regions. In the following, it was considered more convenient to compare relative norm deviations with one another, taking into account the different average values in the respective regions. The relative norm deviations were obtained from division of the norm deviations by the ρ_DFT_ or ρ_val_ 1- and 2-norms of the corresponding reference DFT all-ED and val-ED, respectively, *i.e.* with *m* = 1, 2,

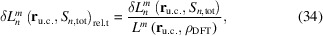




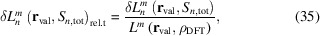




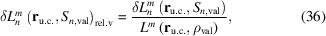




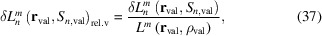

where

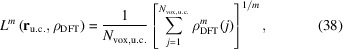




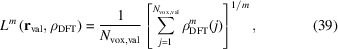




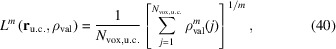




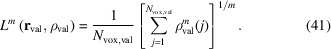

In an analogous way, relative norm deviations for the ED Laplacian distributions were calculated as well [replace in equations (34)–(37) ‘*S_n_*_,DFT_’ and ‘*S_n_*_,val_’ by ‘ 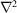 *S_n_*_,DFT_’ and ‘ 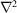 *S_n_*_,val_’, respectively, and in equations (34)–(41) ‘ρ_DFT_’ and ‘ρ_val_’ by ‘ 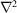 ρ_DFT_’ and ‘ 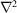 ρ_val_’, respectively].

The 1-norm per voxel of the all-ED ρ_DFT_ has a simple meaning: it is the average ED. Exact summation of the CaB_6_ all-ED ρ_DFT_ in the whole unit cell [equation (38)] would yield *L* ^1^(**r**_u.c._, ρ_DFT_) = 50 e^−^/*V*_u.c._, *i.e.* the average all-ED. Exact summation of the all-ED in the ELI-D valence region [equation (39)] would approximately yield *L* ^1^(**r**_val_, ρ_DFT_) ≃20 e^−^/*V*_u.c._, which is mainly connected with ELI-D shell structure representation reproducing not quantitatively exactly the integer atomic shell occupations given by the periodic table of the elements (Kohout & Savin, 1996 ▸; Baranov, 2014 ▸). Note, while the exact sum (integral) of the ED Laplacian over the unit cell has to be zero due to exact cancellation of positive and negative values, the 1-norm of the ED Laplacian being the sum (integral) of its absolute values is mathematically unbounded from above. The values of the employed *L* ^1^ and *L* ^2^ norms of the reference DFT ED and its Laplacian are given in Table 2 ▸. They all correspond to those obtained by summations on the equidistant grid specified above. Thus, some deviation with respect to the theoretically expected average ED values can be observed, which plays no role in comparison of relative norm deviations of Fourier-synthesized distributions normalized with the same reference DFT ED norm.

Of interest is also the largest absolute deviation of each *S_n_* (**r**) and 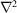 *S_n_* (**r**) distribution in the whole unit cell and in the valence region, denoted according to Δ_max_(**r**_u.c._, *S_n_*_,tot_), Δ_max_(**r**_val_, *S_n_*_,tot_), and Δ_max_(**r**_u.c._, *S_n_*_,val_), Δ_max_ (**r**_val_, *S_n_*_,val_), respectively:

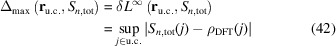




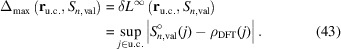

Thus, the following 12 deviation measures for the synthesized ED distributions have been calculated in the present study: δ*L_n_^m^* (**r**_u.c._, *S_n,_*_tot_)_rel.t_, δ*L_n_^m^* (**r**_u.c._, *S_n,_*_val_)_rel.v_, δ*L_n_^m^* (**r**_val_, *S_n_*_,tot_)_rel.t_, δ*L_n_^m^* (**r**_val_, *S_n_*_,val_)_rel.v_, with *m* = 1, 2, and Δ_max_ (**r**_u.c._, *S_n_*_,tot_), Δ_max_ (**r**_val_, *S_n_*_,tot_), Δ_max_ (**r**_u.c._, *S_n_*_,val_) and Δ_max_ (**r**_val_, *S_n_*_,val_). The same 12 quantities are also calculated for the synthesized ED Laplacian distributions: in the formulas replace ‘*S_n_*’ by ‘ 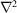 *S_n_*’ and ‘ρ’ by ‘ 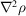 ’.

Further criteria for the quality of the Fourier-synthesized EDs *S_n_* (**r**) are (i) the absence of unphysical negative ED values, and (ii) the absence of NNMs in agreement with the reference ED. The criterion of non-negative ED values was found to be violated by all non-weighted (‘raw’), *i.e.* non-smoothed, Fourier-synthesized all-EDs, a few non-weighted Fourier-synthesized val-EDs and a few Lan1D-synthesized all-EDs with a much too low weighting exponent *p* = 1.0. Application of smoothed Fourier synthesis corresponds in the first step to getting rid of negative EDs. But, as will be shown below, the requirement of the absence of artefact NNMs is a more severe challenge. It plays a major role in evaluating the synthesized ED and ED Laplacian distributions and adjusting the weighting-function exponent accordingly. Roughly speaking, for each Fourier-synthesis method presented herein, the appropriate exponent *p* of the weighting function σ^*p*^(**H** *_j_*) was commonly adjusted (*i.e.* not for each resolution separately) to yield distributions without NNM artefacts for most resolutions, predominantly the higher ones. The choice to keep the *exponent* constant for each *method* was made to allow for studying systematic changes with increasing resolution.

## 3. Results: ED and ED Laplacian distributions from the *method*(*exponent*) type of Fourier synthesis

**Figure 3:**
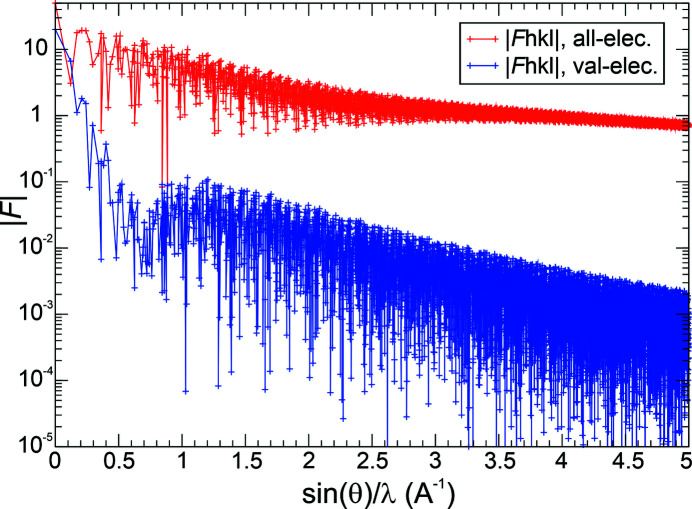
Decay of valence-electron (blue) and all-electron (red) structure-factor amplitudes |*F*(**H**)| for CaB_6_ with increasing resolution.

A look at the structure-factor amplitudes of the all-ED Fourier expansion reveals a very slow decay with increasing resolution (Fig. 3 ▸). From this figure, one would not have a criterion for determining a resolution necessary to yield a sufficiently converged Fourier-synthesized all-electron distribution. The valence-electron structure factors display a local minimum at about [sin(θ)/λ]_max_ ≃ 0.75 Å^−1^, a local maximum at about [sin(θ)/λ]_max_ ≃ 1.3 Å^−1^ and an overall decay that is faster than the all-ED one. Note that the difference between the all-electron and the valence-electron structure factors is the core-electron structure factors (not shown). For the val-ED structure factors, the decrease of one order of magnitude from the local maximum at ∼0.75 Å^−1^ is only achieved at ∼3.25 Å^−1^, which would be too high for experimental studies. The situation is even worse for the all-ED structure factors.

For raw Fourier synthesis, the similar size of neighbouring structure factors increases the possibility of sudden topological changes on inclusion of further neighbours, *i.e.* a non-convergence of topological features, which is the typically observed behaviour. This can be prevented using smoothing factors σ^*p*^(**H** *_j_*) as described above. In the following, the raw Fourier synthesis, which corresponds to constant weights σ^*p*^(**H** *_j_*) = 1 for all structure factors *F*(**H** *_j_*), will be considered the most aggressive weighting scheme compared with all other ones with smaller and variable weights. As will be seen below, raw Fourier synthesis with constant weights [equation (3)] has a strong tendency towards creation of artificial NNMs, and even regions with negative ED values upon Fourier-series truncation.

**Figure 4:**
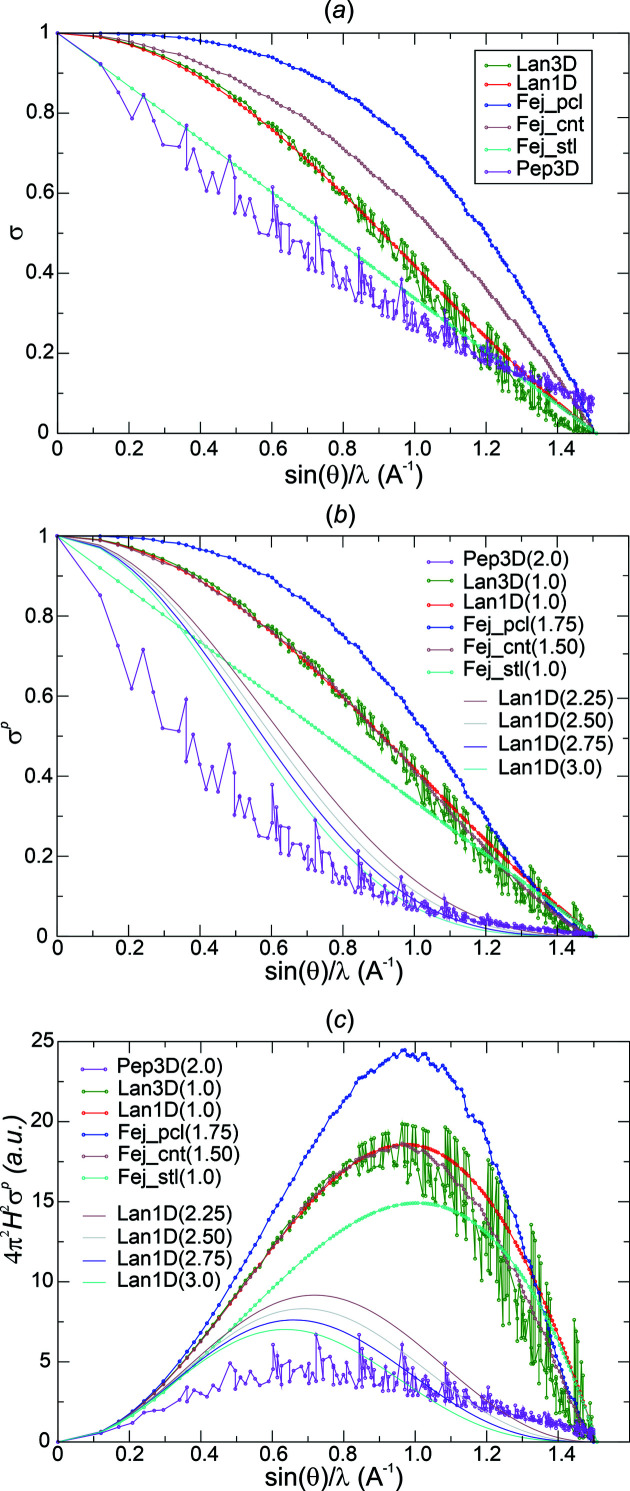
Decay of types of weighting function σ*^p^* used, shown for [sin(θ)/λ]_max_ = 1.5 Å^−1^: (*a*) principal decay, *p* = 1; (*b*) smoothing used with various exponents 1.0 ≤ *p* ≤ 3.0; (*c*) complete prefactor (2π*H_j_*)^2^σ*^p^* (**H** *_j_*) used for ED Laplacian synthesis. To distinguish the Lan1D smoothing curves for all-ED synthesis (2.25 ≤ *p* ≤ 3.0) from the val-ED ones (*p* = 1.0), they are always shown as pure lines skipping the data points.

With *exponent p* = 1, overall concave decay (negative curvature) of σ*^p^* (*H_j_*) is found for all 1D Fejer-type methods, except Fej_stl with linear decay throughout the whole range [0, [sin(θ)/λ]_max_] [Fig. 4 ▸(*a*)]. In contrast, the 3D Fejer-type Pep3D factors on average display an overall convex decay. The Lanczos-derived schemes yield an initially concave decay, but exhibit an inflection point towards the end of the interval and become slightly convex. The most aggressive weighting schemes are found to be the Fejer-type ones Fej_pcl and Fej_cnt, which display the highest weights for most of the resolution interval [0, [sin(θ)/λ]_max_]. In particular, for these methods, NNMs were found for several resolutions (Table 3 ▸). This behaviour was counteracted by increasing the exponent *p* until a reasonable amount of resolutions became free of NNM artefacts at ½*H*_max_ ≥ 1.1 Å^−1^. This was the case at values *p* = 1.5 and 1.75 for Fej_cnt and Fej_pcl, respectively [Fig. 4 ▸(*b*)]. The less aggressive Lanczos-derived weights did not yield NNMs for ½*H*_max_ ≥ 1.1 Å^−1^ at *p* = 1.0 for the val-ED structure factors, but for the smoothing of all-electron structure-factor synthesized all-EDs with the Lan1D method, *p* = 2.25, 2.50, 2.75 and 3.0 had to be employed. In general, exponentiation with *p* > 1.0 increases the initial decay of σ*^p^* (*H*_j_) and introduces a concave tail region or increases the degree of concavity of the tail region.

With the initially (½*H*_max_ ≃ 0) convex and tailing concave shape, the sigma factors σ^*p*^(*H*_j_) are similar to the Debye–Waller factor which decreases according to exp(−¼*B*_iso_ *H_j_* ^2^). It is also interesting to see how the completely different methods Lan1D and Fej_cnt showing clearly different weighting factors σ^*p*^ for *p* = 1.0 [Fig. 4 ▸(*a*)] accidentally obtain very similar weights upon using the finally adjusted Fej_cnt exponent of *p* = 1.5 [Fig. 4 ▸(*b*)]. From a signal processing point of view, the σ^*p*^ factors for Fourier synthesis of the ED act as a low-pass filter. For the Fourier synthesis of smoothed ED Laplacian distributions, the σ^*p*^ factors play a decisive role by over-compensating the quadratically increasing prefactor (2π*H_j_*)^2^, which would lead to non-convergence of the raw Fourier series. For the ED Laplacian reconstruction, the σ^*p*^ factors act as a bandpass filter [Fig. 4 ▸(*c*)].

### 3.1. Fourier synthesis of val-ED and val-ED Laplacian distributions

Fourier synthesis [equations (15), (16)] of val-ED *S_n_*_,val_(**r**) and val-ED Laplacian distributions 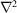 *S_n_*_,val_(**r**) employs static val-ED structure factors. In view of the intended QTAIM analysis, the derived [equations (19), (20)] all-electron distributions 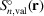 and 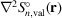 with added core distributions of the reference DFT wavefunction are always analysed in the following. The difference between these all-electron distributions and the corresponding reference DFT distribution is equal to the difference between the corresponding val-ED and val-ED Laplacian distributions [equations (21), (22)].

Raw Fourier synthesis of val-ED without smoothing [equation (3)] results in NNMs even at resolution ½*H*_max_ = 1.5 Å^−1^ [Fig. 2 ▸(*b*)]. Therefore, for each method employed the exponent *p* of the smoothing factor σ^*p*^(**H**) was increased stepwise until a suitable number of resolutions in the range 0.5 Å^−1^ ≤ ½*H*_max_ ≤ 5.0 Å^−1^ were free of NNMs. In order to study the important systematic changes for each method, the exponent *p* was not adjusted for each resolution separately. As a result, for each method a number of NNMs occurring at lower resolutions were accepted (Table 3 ▸).

**Figure 5:**
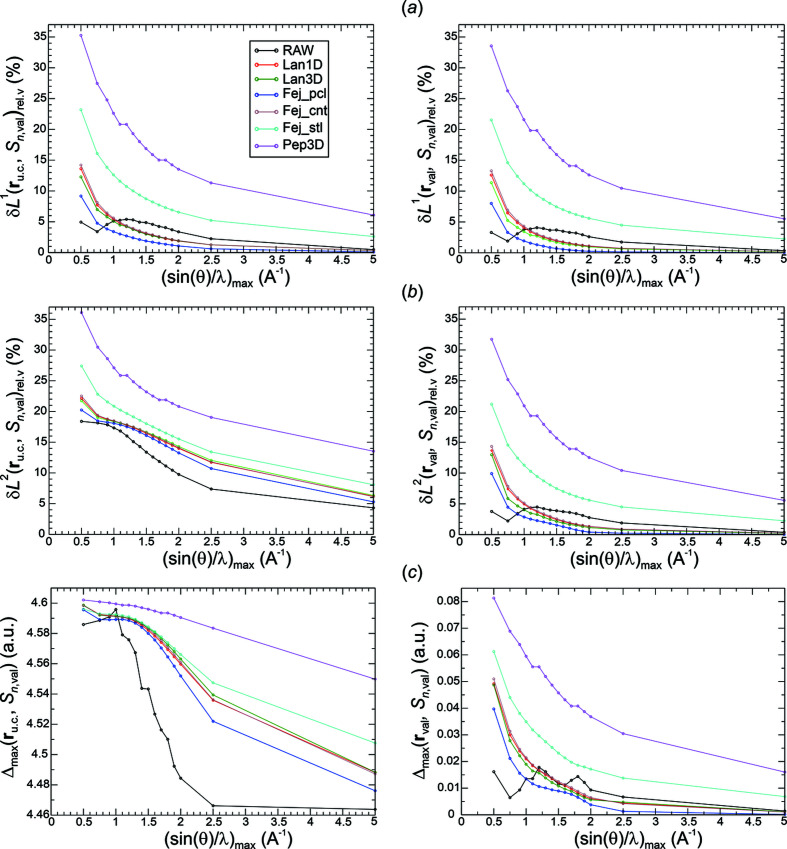
Norm deviations for Fourier synthesis of val-ED *S_n_*_,val_(**r**) for methods Lan1D(1.0), Lan3D(1.0), Fej_pcl(1.75), Fej_cnt(1.5), Fej_stl(1.0) and Pep3D(2.0) [for colour coding, see legend in top-left figure (*a*)] and different resolutions [sin(θ)/λ]_max_. (*a*), (*b*), (*c*) Norm deviations δ*L*_*n*_^1^, δ*L*_*n*_^2^ and Δ_max_, respectively; left panels, complete unit-cell region **r**_u.c._; right panels, valence region **r**_val_.

*Analysis of val-ED norm deviations*. Raw Fourier synthesis of val-ED distributions is expected to converge in the δ*L*_*n*_^2^ norm, which is depicted in the left panel of Fig. 5 ▸(*b*). The δ*L*_*n*_^2^ deviations for the raw synthesis are always smaller than the ones for the smoothed Fourier syntheses. The opposite behaviour is found for the δ*L*_*n*_^1^ deviations at higher resolutions [except Pep3D(2.0*)*, Fej_stl(1.0)], where the raw synthesis even displays a local maximum at about 1.2 Å^−1^ [Fig. 5 ▸(*a*), left panel]. The different behaviour of the raw and smoothed ED distributions indicated by δ*L*_*n*_^1^ and δ*L*_*n*_^2^ norms is a result of the raw Fourier synthesis improving more on the higher deviations than the smoothed ones. This can be seen from the maximum norms given in the left panel of Fig. 5 ▸(*c*). The monotonic decay of the δ*L*_*n*_^1^ norms for the smoothed Fourier syntheses is the mathematically expected behaviour. It is related to the expected uniform convergence for these weighting functions *methods*(*p*) as shown by the monotonic decay of the corresponding maximum norms in the left panel of Fig. 5 ▸(*c*). The overall decay of the maximum norm for the raw Fourier synthesis and the convergence indicated at higher resolutions indicate an unexpected uniform convergence of the raw val-ED Fourier synthesis in the full region.

The norm deviations in the valence region **r**_val_, shown in the right panels of Figs. 5 ▸(*a*)–5 ▸(*c*), are always smaller than the corresponding ones in the total region **r**_u.c._ [Figs. 5 ▸(*a*)–5 ▸(*c*), left panels], but to a variable degree. Notably, the uniform convergence for the smoothed synthesis is also found to be valid for the valence region [Figs. 5 ▸(*a*), 5 ▸(*c*), right panels], which was not initially expected.

**Figure 6:**
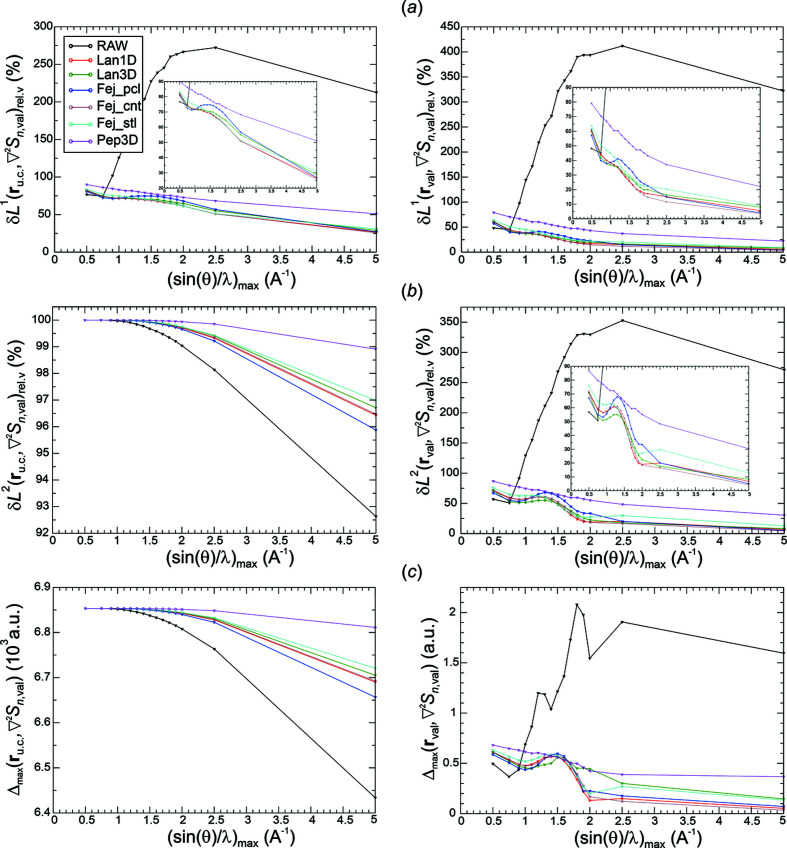
Norm deviations for Fourier synthesis of val-ED Laplacian 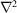 *S_n_*_,val_(**r**) for *methods*(*p*) Lan1D(1.0), Lan3D(1.0), Fej_pcl(1.75), Fej_cnt(1.5), Fej_stl(1.0) and Pep3D(2.0) [for colour coding, see legend in top-left figure (*a*)] and different resolutions [sin(θ)/λ]_max_. (*a*), (*b*), (*c*) Norm deviations δ*L*_*n*_^1^, δ*L*_*n*_^2^ and Δ_max_, respectively; left panels, complete unit-cell region **r**_u.c._; right panels, valence region **r**_val_.

*Analysis of val-ED Laplacian norm deviations*. The δ*L*_*n*_^2^ deviations of the synthesized val-ED Laplacian distributions in the full region [Fig. 6 ▸(*b*), left panel] display a monotonic decay of the raw and the smoothed distributions. The corresponding δ*L*_*n*_^1^ [Fig. 6 ▸(*a*), left panel] and maximum norms Δ_max_ [Fig. 6 ▸(*c*), left panel] reveal the uniform convergence of the smoothed and even the raw val-ED Laplacian distributions in the full region.

In the valence region, the deviations δ*L_n_* ^1^, δ*L_n_* ^2^ and Δ_max_ [Figs. 6 ▸(*a*)–6 ▸(*c*), right panels] of the synthesized val-ED Laplacian feature partially non-monotonic decays at lower resolutions and uniform convergence at higher resolutions for all functions [very slowly for Pep3D(2.0)]. The observed monotonic decays of the δ*L_n_* ^1^ norms in the valence region found for all smoothing functions besides the Fej_pcl(1.75) one may also suggest a certain regular convergence for the critical point Laplacian values. However, as can be seen in the critical points section below, all methods (with their specific exponents *p* used here) besides Pep3D(2.0) and Lan3D(1.0) display a similar damped oscillatory convergence behaviour with respect to deviation from the reference critical point values.

**Figure 7:**
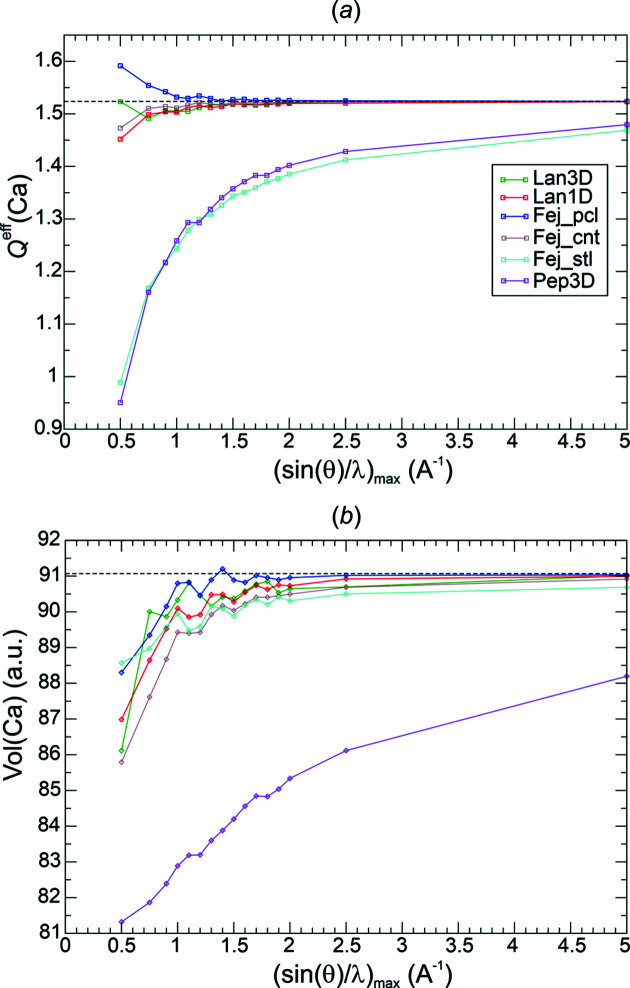
QTAIM analysis of all-ED 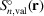 obtained from Fourier synthesis of val-ED of CaB_6_ with subsequently added reference core-ED [equation (19)] using *methods*(*p*) Lan3D(1.0), Lan1D(1.0), Fej_pcl(1.75), Fej_cnt(1.5), Fej_stl(1.0) and Pep3D(2.0); (*a*) QTAIM effective charges *Q* ^eff^(Ca), and (*b*) associated atomic volumes Vol(Ca); dashed horizontal lines indicate values obtained from the reference DFT ED.

*QTAIM basin analysis of* 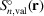 . Classical QTAIM topological analysis was performed on the all-ED 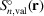 obtained by adding the correct core-ED of the reference DFT calculation to the synthesized val-ED [equation (19)]. With two crystallographically distinct atom types, it is sufficient to display the results for the Ca atomic basin only with a reference DFT value of *Q* ^eff^(Ca) = +1.52. The numerical recovery of the total electron number 50 e^−^/u.c. showed an error of maximally 0.0002 electrons in all investigations. The occurrence of NNMs for some resolutions (Table 2 ▸) does not lead to visible effects in the basin population and volume curves shown (Fig. 7 ▸).

All weighting functions *methods*(*p*) investigated display a rather regular convergence of the QTAIM atomic charges with increasing resolution [Fig. 7 ▸(*a*)]. For all functions except Fej_stl(1.0) and Pep3D(2.0), a chemically reasonable accuracy was obtained at resolutions beyond 0.75 Å^−1^. With respect to the decay of the weighting factors [Fig. 4 ▸(*b*)], these functions correspond to the least aggressive ones. They are found to display the larger ED norm deviations (Fig. 5 ▸). Interestingly, the Ca atomic volumes [Fig. 7 ▸(*b*)] do not simply display a similar behaviour to the charge curves, which is most clearly seen for function Fej_stl(1.0). Despite the comparably bad charge reconstruction, its volume reconstruction is comparable with the more competitive functions.

**Figure 8:**
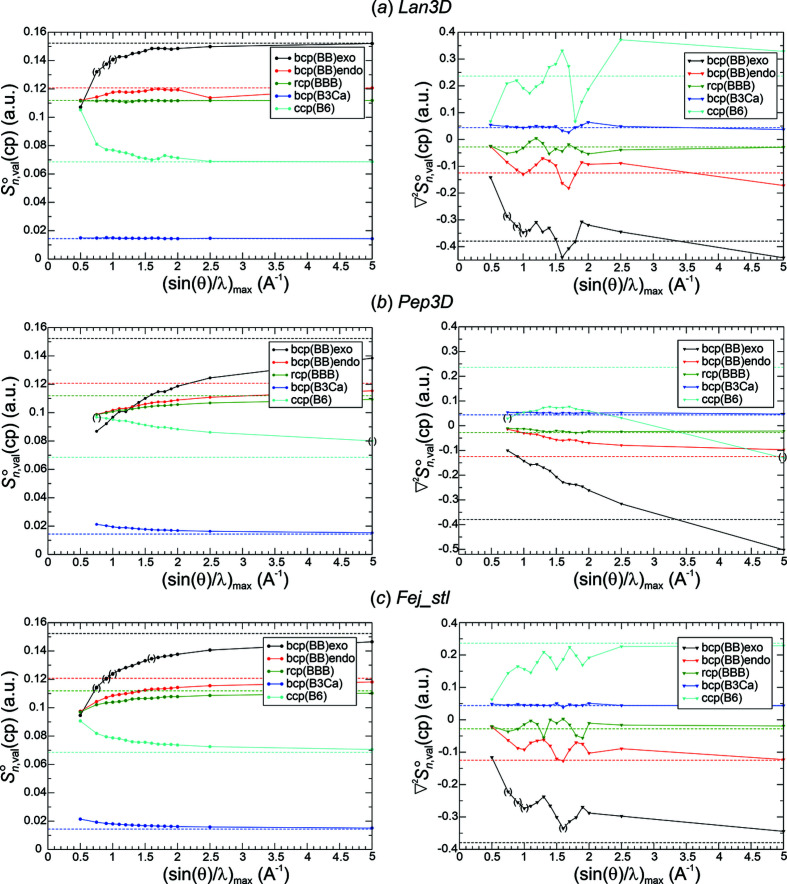
Critical point evaluation for all-EDs 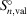 (**r**_c.p._) (right panel) and all-ED Laplacians 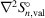 (**r**_c.p._) (left panel) obtained from val-ED and val-ED Laplacian [equations (19), (20)] Fourier synthesis using weighting functions (*a*) Lan3D(1.0), (*b*) Pep3D(2.0) and (*c*) Fej_stl(1.0). Dashed horizontal lines indicate values obtained from the reference DFT ED; data symbols in brackets ‘()’ indicate values at position (½, ½, 0) of original b.c.p.(BB)_exo_, where an NNM is found (Table 3 ▸).

**Figure 9:**
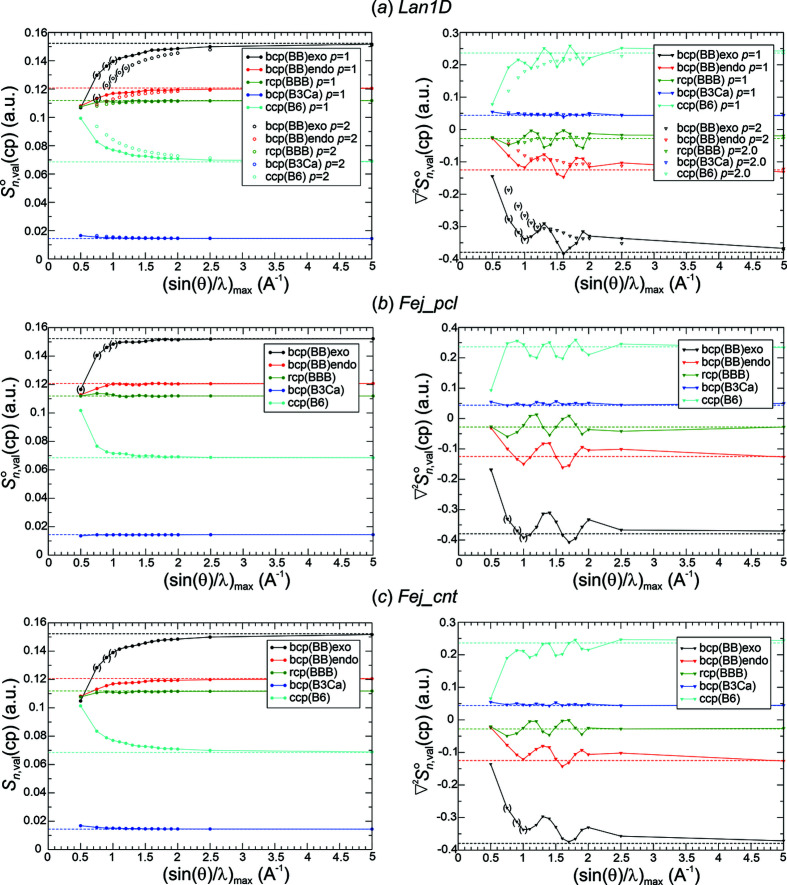
Critical point evaluation for all-EDs 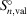 (**r**_c.p._) (right panel) and all-ED Laplacians 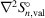 (**r**_c.p._) (left panel) obtained from val-ED and val-ED Laplacian [equations (19), (20)] Fourier synthesis using weighting functions (*a*) Lan1D(1.0, 2.0), (*b*) Fej_pcl (1.75) and (*c*) Fej_cnt(1.5). Dashed horizontal lines indicate values obtained from the reference DFT ED; data symbols in brackets ‘()’ indicate values at position (½, ½, 0) of original b.c.p.(BB)_exo_, where an NNM is found (Table 3 ▸).

*Critical point analysis of* 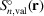 . The most challenging quality aspect for the Fourier-synthesized all-ED 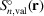 [equation (19)] and all-ED Laplacian 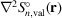 [equation (20)] distributions is their ability to reproduce the chemical content contained in the original DFT-calculated distribution. For this purpose, critical points for all Fourier-synthesized ED distributions have been determined and analysed. The chemical focus requires (based on the DFT ED and chemical bonding arguments) the increase of ED at critical points in the sequence Γ_8_(c.c.p.-B_6_) < Γ_6_(r.c.p.-BBB) < Γ_4_(b.c.p.-BB_endo_) < Γ_5_(b.c.p.-BB_exo_), negative values of the ED Laplacian for Γ_4_(b.c.p.-BB_endo_), Γ_5_(b.c.p.-BB_exo_), Γ_6_(r.c.p.-BBB), and positive ones for Γ_3_(b.c.p.-B_3_Ca) and Γ_8_(c.c.p.-B_6_) increasing according to Γ_5_ < Γ_4_ < Γ_6_ < Γ_3_ < Γ_8_ (*cf*. Fig. 1 ▸). These relations of signs and values are found to already be obeyed at lower resolutions for all Fourier-synthesis weighting functions *methods*(*p*) except for Pep3D(2.0) (Figs. 8 ▸, 9 ▸), even in those cases where at the original position of Γ_5_(b.c.p.-BB_exo_) an NNM has been found. A rather monotonic approach of the corresponding ED values towards the initial ones is observed for all these functions (Figs. 8 ▸, 9 ▸, left panels). The Pep3D(2.0) function [Fig. 8 ▸(*b*)] displays the largest deviations and slowest convergence of all functions. Concerning the ED Laplacian (Figs. 8 ▸, 9 ▸, right panels), none of the functions features a monotonic decrease of the deviations with respect to the original values. Instead, a rather similar oscillating approach is observed for most of them. This can be remedied by an increase of the smoothing factor’s exponent *p*. For method Lan1D this was tested by increasing from initially *p* = 1.0 to *p* = 2.0 [Fig. 9 ▸(*a*), right panel]. It can be seen that the oscillatory approach to the reference values can be largely smoothed out, but at the price of systematically increased ED deviations [Fig. 9 ▸(*a*), left panel]. A wider discussion of this issue is found in the next section.

Weighting function Pep3D(2.0) even seems to fail converging the ED Laplacian at the critical points investigated [Fig. 8 ▸(*b*), right panel]. Searching for an indication of this failure in the norm deviations, it is striking that the maximum norm in the valence region does not seem to converge at all [Fig. 6 ▸(*c*), right panel]. On the other hand, Lan3D(1.0) also seems to have a problem converging the ED Laplacian at the critical points [Fig. 8 ▸(*a*), right panel], but its convergence of the maximum norm in the valence region does not look unusual [Fig. 6 ▸(*c*), right panel]. Whether this can be remedied by increasing the smoothing factor exponent, similar to the Lan1D case, has not been tested.

Another important point concerns the size of the deviations at each critical point **r**_c.p._ for a given resolution. It is already obvious from the figures (Figs. 8 ▸, 9 ▸) that the absolute deviations of the synthesized ED and ED Laplacian values with respect to the reference values at each type of critical point are rather different. This observation is also valid for the relative deviations, given by

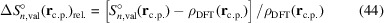

and

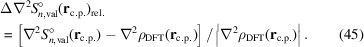




As an example, a collection of absolute and relative deviations from Lan1D(1.0, 2.0) Fourier synthesis are given for resolutions of 1.1 and 1.3 Å^−1^ in Table 4 ▸. The results for Lan1D(1.0) at ½*H*_max_ = 1.1 Å^−1^ could be considered as the low-resolution limit of a successful Fourier synthesis with method Lan1D being free from NNMs. However, within this series of Fourier syntheses the oscillatory approach to the reference values and visual ED Laplacian contour ripples (see the next section) for the higher resolutions (not yet for this comparably low resolution) may be disturbing. Certainly, the exceptionally high relative deviation of the ED Laplacian at r.c.p.-BBB of +87.7% is connected with this feature. A deviation of +100% would make the Laplacian value equal to 0, which would be a failure to qualitatively reproduce the chemical bonding condition 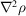 (r.c.p.-BBB) < 0. An alternative candidate is Lan1D(2.0) at ½*H*_max_ = 1.3 Å^−1^, where a lower resolution cannot be chosen because of NNM occurrence. It has the advantage of being a member of the Lan1D(2.0) series with a more regular convergence behaviour and negligible ED Laplacian ripples (see the next section) not only for this resolution, but also for higher ones up to 5.0 Å^−1^. Obviously, there is not one best choice of low-resolution limit, it rather depends on priorities set up by the requirements of the investigation. This is also valid for the other methods presented (Figs. 8 ▸, 9 ▸).

**Figure 10:**
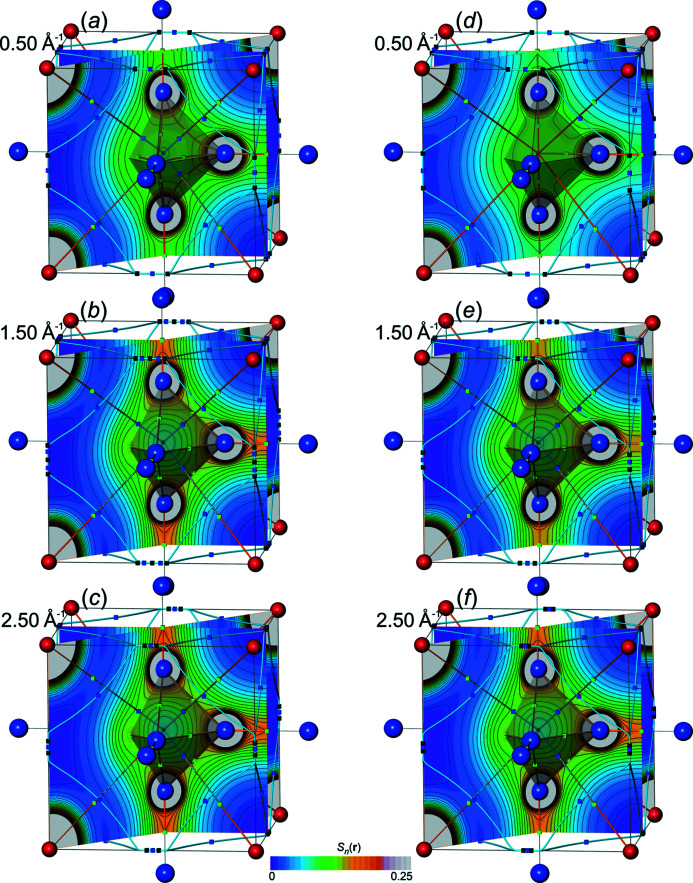
Resolution-dependent all-ED distributions 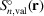 in CaB_6_ from val-ED synthesis [equation (19)] for each resolution indicated using method Lan1D. (*a*)–(*c*) employing exponent *p* = 1.0; (*d*)–(*f*) *p* = 2.75. The molecular graphs with b.c.p.’s (green), r.c.p.’s (blue) and c.c.p.’s (black) are shown; isolines are drawn at intervals of 0.01 e^−^ bohr^−3^. Note that the topology of (*d*) is substantially different from all the other ones: there is an NNM at the octahedron centre, and b.c.p.(B–B_endo_) and r.c.p.(B_3_) are missing.

**Figure 11:**
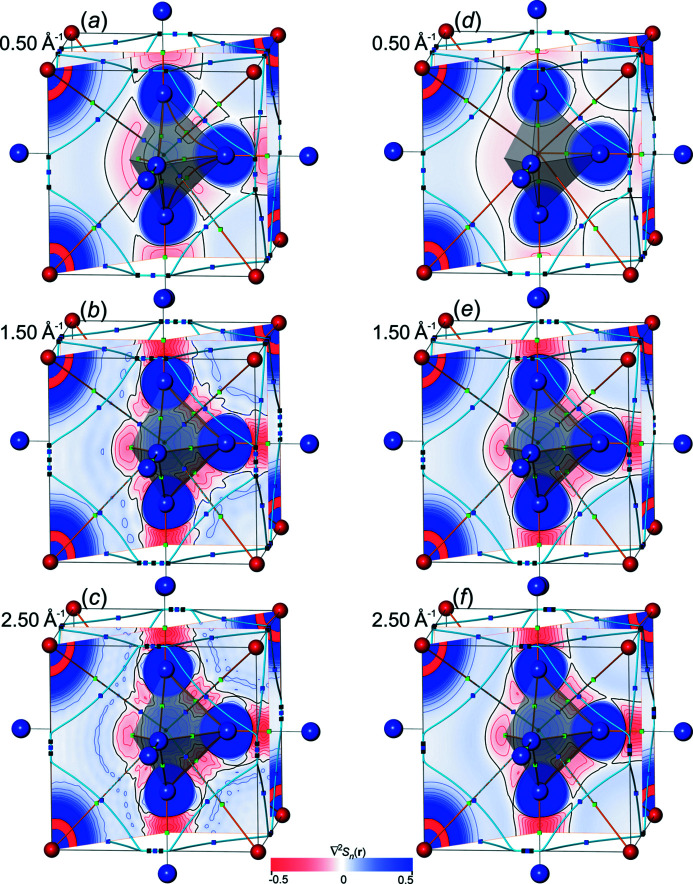
Resolution-dependent all-ED Laplacian distributions 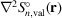 in CaB_6_ from val-ED Laplacian synthesis [equation (20)] for each resolution using method Lan1D. (*a*)–(*c*) employing exponent *p* = 1.0; (*d*)–(*f*) *p* = 2.75. The molecular graphs with b.c.p.’s (green), r.c.p.’s (blue), and c.c.p.’s (black) are shown; isolines are drawn at intervals of 0.05 e^−^ bohr^−5^. Note that the topology of (*d*) is substantially different from all the other ones: there is an NNM at the octahedron centre, and b.c.p.-BB_endo_ and r.c.p.-B_3_ are missing.

**Figure 12:**
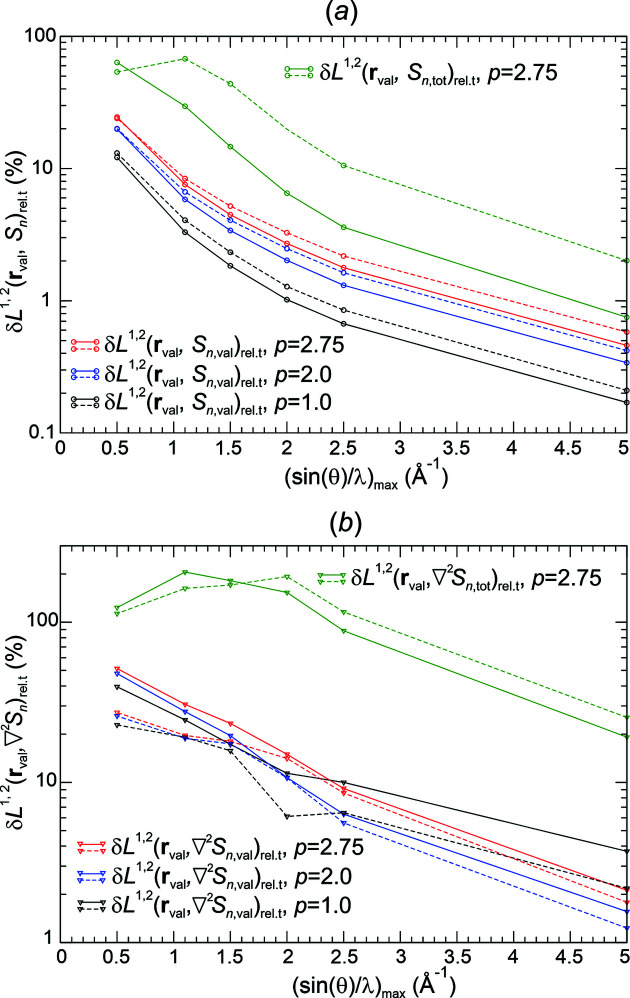
CaB_6_: valence region relative norm deviations δ*L*_*n*_^1^ (full lines) and δ*L*_*n*_^2^ (dashed lines) for Lan1D synthesis of (*a*) val-ED *S_n_*_,val_(**r**) and all-ED *S_n_*_,tot_(**r**), and (*b*) val-ED Laplacian 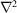 *S_n,_*_val_(**r**) and all-ED Laplacian 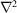 *S_n_*_,tot_(**r**). Val-ED and val-ED Laplacian synthesis with exponents *p* = 1.0 (black lines), 2.0 (blue lines), 2.75 (red lines), and all-ED and all-ED Laplacian synthesis with *p* = 2.75 (green lines).

*All-ED* 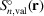 *and all-ED Laplacian* 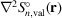 *distributions*. In order to get a visual impression for the all-ED 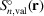 and all-ED Laplacian distributions 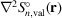 obtained [equations (19), (20)] from Fourier-synthesized val-ED *S_n_*_,val_(**r**) and val-ED Laplacian 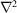 *S_n_*_,val_(**r**), a selection of corresponding all-ED and all-ED Laplacian maps is presented in Figs. 10 ▸ and 11 ▸, respectively. For comparison with the Fourier-synthesized all-ED and all-ED Laplacian distributions (see below), those obtained with method Lan1D have been chosen here as well. For this method the value *p* = 1.0 of the weighting-function exponent could be selected for val-ED synthesis, which led to non-occurrence of NNMs for ½*H*_max_ ≥ 1.1 Å^−1^ and for ½*H*_max_ = 0.5 Å^−1^. All synthesized ED distributions are completely smooth (Fig. 10 ▸), in contrast to the more challenging ED Laplacian distributions (Fig. 11 ▸, left column). It can be seen that increasing resolution ½*H*_max_ is accompanied by increasing amount of contour ripples in the ED Laplacian distributions, which is a consequence of the rather aggressive weighting (*i.e.* comparably large weights). For illustration of the effect of less aggressive weighting (smaller weights) on the Laplacian distribution smoothness, a comparison with a higher smoothing exponent *p* = 2.75 is shown in Fig. 11 ▸, right column. It can be seen that with *p* = 2.75 the ED Laplacian contour ripples can be smoothed out. No systematic efforts have been undertaken to find for each resolution the smallest necessary exponent *p* value for obtaining a certain predefined smoothness of the ED Laplacian contours. It may be sufficient to mention that already with *p* = 2.0 all ED Laplacian contours calculated with this model are visually smooth (a numerical criterion has not been employed). The value *p* = 2.75 was chosen here to make a visual comparison with the Fourier-synthesized all-ED distributions shown below, which have been obtained with the same exponent. For the synthesized val-ED distributions, the higher exponents *p* = 2.0 and 2.75 yield systematically higher overall δ*L*_*n*_^1^ and δ*L*_*n*_^2^ norm deviations of the val-ED in valence regions than with more aggressive weighting *p* = 1.0 [Fig. 12 ▸(*a*)].

For the synthesized val-ED Laplacian the situation is more complex. The δ*L*_*n*_^1^(**r**_val_, 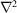 *S_n,_*_val_) deviations in the valence region [Fig. 12 ▸(*b*)] are found to be smaller with aggressive weighting *p* = 1.0 than with *p* = 2.75 weighting until ½*H*_max_ = 2.0 Å^−1^, and become larger for resolutions ½*H*_max_ ≥ 2.5 Å^−1^. For all resolutions shown [Fig. 12 ▸(*b*)], the intermediate exponent *p* = 2.0 yields lower norm deviations δ*L*_*n*_^1,2^ than the *p* = 2.75 one. Thus, for higher resolutions ½*H*_max_ ≥ 1.5 Å^−1^, an exponent 1.0 < *p* ≤ 2.0 Å^−1^ might be sufficient to prevent visible Laplacian contour ripples. This may be related to the original Lanczos result (Lanczos, 1956 ▸) of *p* = 1 for gradient synthesis, implying *p* = 2 for ED Laplacian synthesis. It is, however, not clear how his results on purely 1D Fourier synthesis apply to a 1D condensed variant (like Lan1D) of a real 3D Fourier-synthesis task. In summary, it seems that the increasing rippling in the ED Laplacian distribution contours has an adverse effect on the δ*L*_*n*_^1^ norms of the *p* = 1.0 syntheses, and that they improve upon smoothing out those ripples. Once the ripples are smoothed out (with *p* = 2.0), the norm deviations become worse again upon increased smoothing (*p* = 2.75).

Certainly, the smoothing of ED Laplacian contours via increased *p* values simultaneously increases the ED δ*L*_*n*_^1^ deviations [seen also locally in critical point analysis, Fig. 9 ▸(*a*), left panel] of the already smooth ED distributions as just indicated, which means that a suitable compromise *p* value must be found, depending on the focus of the application.

### 3.2. Fourier synthesis of all-ED and all-ED Laplacian distributions

As was shown in Fig. 3 ▸, the all-ED structure factors decay very slowly with increasing resolution ½*H*_max_, which certainly makes Fourier synthesis in the atomic core regions a very challenging task. However, the question arises: to what extent these effects adversely affect ED analysis in the valence region? Because of its quality in reproduction of the chemical bonding features with exponent *p* = 1.0 for val-ED Fourier synthesis, method Lan1D was chosen to investigate this issue. By systematic *p*-value variation it was found that for all-ED Fourier synthesis, exponents in the range 2.25 ≤ *p* ≤ 3.0 are useful to prevent negative ED and NNM artefacts for most resolutions used (Table 5 ▸). The preferred model for systematic evaluations was the Lan1D(2.75) one, which showed no such artefacts for all resolutions. Other smoothing variants in the indicated range were also investigated, because for certain resolutions, *i.e.* ½*H*_max_ = 1.5 Å^−1^, their Fourier syntheses were also free of artefacts, and therefore competitive alternatives to the *p* = 2.75 variant.

**Figure 13:**
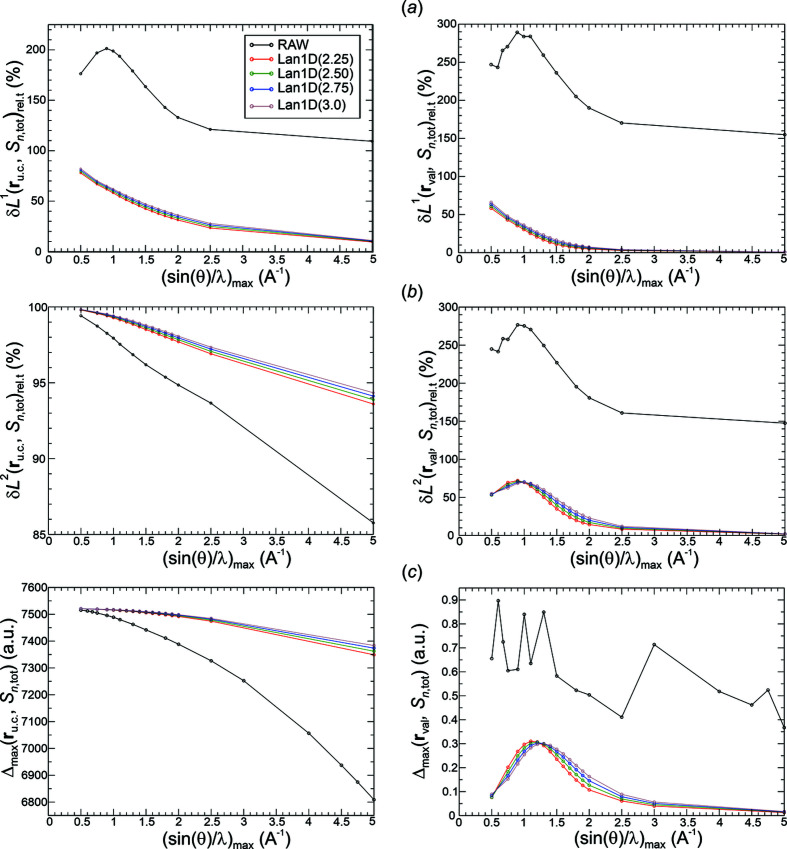
Fourier synthesis of all-ED for CaB_6_ using method Lan1D with exponents *p* = 2.25, 2.50, 2.75 and 3.0. (*a*)–(*c*) Norm deviations δ*L*_*n*_^1^, δ*L*_*n*_^2^ and Δ_max_, respectively, of all-ED *S_n_*_,tot_(**r**); left panel, total u.c. region **r**_u.c._; right panel, valence region **r**_val_.

*Analysis of all-ED norm deviations*. Raw Fourier synthesis of the all-ED is expected to converge in the δ*L*_*n*_^2^ norm, which is verified in the left panel of Fig. 13 ▸(*b*). The δ*L*_*n*_^2^ deviations are smaller than the ones for the smoothed Fourier syntheses, and the convergence is faster. The opposite behaviour is found for the δ*L*_*n*_^1^ deviations depicted in the left panel of Fig. 13 ▸(*a*). Here, the smoothed all-ED distributions have lower deviations than the raw one, which is a result of the raw Fourier synthesis improving more on the higher deviations occurring at higher values [Fig. 13 ▸(*c*), left panel], namely in the core regions. The raw Fourier-synthesis δ*L*_*n*_^1^ deviations display the occurrence of a local maximum at about 0.9 Å^−1^ and convergence at higher resolutions. The convergence of the δ*L*_*n*_^1^ norm for the smoothed Fourier syntheses [Fig. 13 ▸(*a*), left panel] is the mathematically expected behaviour. It is related to the uniform convergence obtained using these weighting functions *methods*(*p*) as can be seen by the monotonic decay of the corresponding maximum norms in the left panel of Fig. 13 ▸(*c*). The monotonic decay of the maximum norm on the grid for the raw Fourier synthesis indicates uniform convergence as well, which is surprising and the detailed reason is not known. Certainly, for all resolutions investigated, the maximum norm is found at the position of the Ca nucleus, which is located on a grid point. This can be considered a lucky circumstance, because it is the position where the maximum norm over the whole unit-cell region is expected to be located, mainly because the boron nucleus (not being located on the evaluation grid) has a lower atomic number. It is important to realize that with large deviations from the reference DFT ED at the Ca nuclear position and their slow convergence indicated by values of ρ_DFT_(**r**_Ca_) ≃ 7522 bohr^−3^ and *S_n_*_,tot_(**r**_Ca_) ≃ 713 bohr^−3^ for raw synthesis and 148 bohr^−3^ for Lan1D(2.75) at ½*H*_max_ = 5.0 Å^−1^, Fourier synthesis of the all-ED in the core region near the nucleus is not suitable in a quantitative way with the *method*(*exponent*) type of approach applied here. The question remains as to whether all-ED Fourier synthesis in the valence region can give chemically meaningful results.

In the valence region, all deviation types δ*L*_*n*_^1^, δ*L*_*n*_^2^ and Δ_max_ [Figs. 13 ▸(*a*)–13 ▸(*c*), right panels] for the smoothed all-ED distributions are much smaller than for the corresponding raw ones. While the maximum norm of the raw distribution displays an irregular behaviour up to high resolutions, the smoothed ones decay monotonically beyond a local maximum at about 1.2 Å^−1^. It is noteworthy that the locations of the maximum norm in the valence region are always found at grid points directly in the neighbourhood of the exclusion radius of the boron atoms, such that their values are considered to be (and actually found to be, see below) significantly larger than the deviations expected at the critical point locations deep inside the valence region. Although overall monotonic decay of δ*L*_*n*_^1^ and δ*L*_*n*_^2^ in the valence region is not seen for the smoothed distributions, uniform convergence of Fourier-synthesized all-EDs is indicated by the norms δ*L_n_* ^1^, δ*L_n_* ^2^ and Δ_max_ at higher resolutions [Figs. 13 ▸(*a*)–13 ▸(*c*), right panels]. The more aggressive weighting functions (lower *p* values, higher weights) yield the smaller deviations at these resolutions.

**Figure 14:**
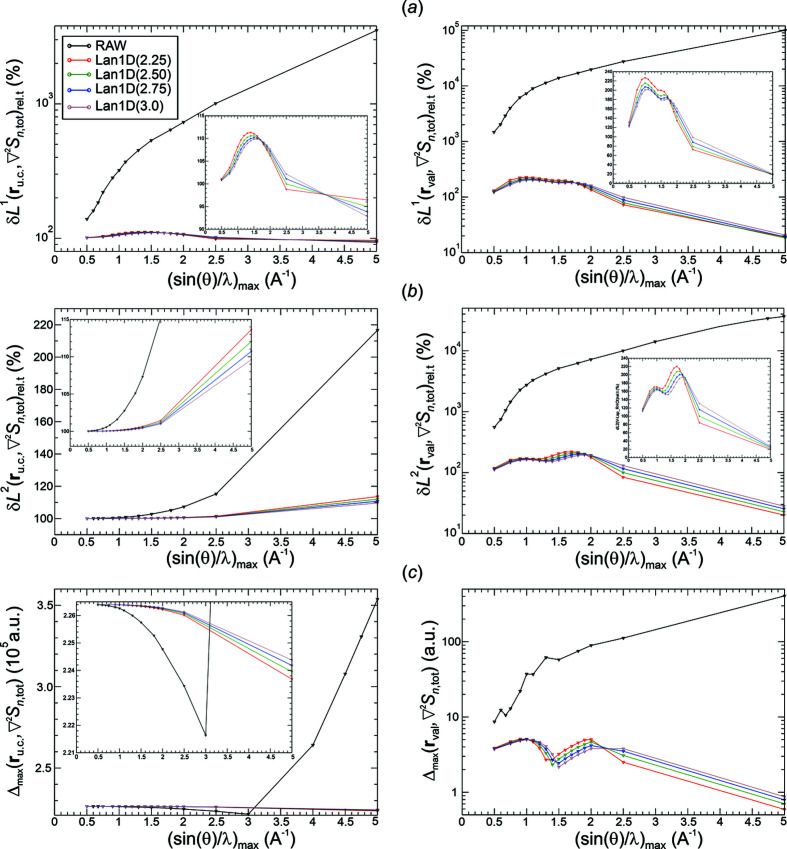
Fourier synthesis of all-ED Laplacian for CaB_6_ using method Lan1D with exponents *p* = 2.25, 2.50, 2.75 and 3.0. (*a*)–(*c*) Norm deviations δ*L*_*n*_^1^, δ*L*_*n*_^2^ and Δ_max_, respectively, of all-ED Laplacian 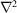 *S_n_*_,tot_(**r**); left panel, total u.c. region **r**_u.c._; right panel, valence region **r**_val_.

*Analysis of all-ED Laplacian norm deviations*. For raw Fourier synthesis the all-ED Laplacian deviations in the full unit-cell region are divergent [Figs. 14 ▸(*a*)–14 ▸(*c*), left panel] as mathematically expected. While this is clearly indicated in the δ*L*_*n*_^1^ and δ*L*_*n*_^2^ norm deviations [Figs. 14 ▸(*a*), 14 ▸(*b*), left panel], convergent behaviour of the maximum norm Δ_max_ is simulated at lower resolutions until a sudden strong increase beyond 3.0 Å^−1^ changes the convergence scenario [Fig. 14 ▸(*c*), left panel]. The abrupt rise is an artefact caused by the combination of two factors, namely (i) the finite grid resolution, and (ii) the electron–nuclear cusp (Kato, 1957 ▸). Due to the shape of the plane waves, the electron–nuclear cusp cannot be represented by Fourier synthesis with structure factors available at any experimental conditions and not in the present theoretical study either. In contrast, the electron–nuclear cusp is present in the given reference DFT all-ED of CaB_6_ and leads to a value of 0 for the DFT all-ED Laplacian given at the nuclear positions (in fact, the value is mathematically undefined, and is arbitrarily given a value of 0), such that these positions were excluded from the maximum norm evaluations of the synthesized all-ED Laplacian distributions. On the regular grid used, this only concerns the Ca position at (0, 0, 0), which is a grid point, while the B-atom positions are not located on grid points. Thus, on evaluation of the full unit-cell maximum norm of the all-ED Laplacian, the highest deviation is found for a grid point closest to the B nucleus for all resolutions ½*H*_max_ ≤ 3.0 Å^−1^. For all higher resolutions investigated, another grid point features the highest deviation. It is located one grid point away from the Ca position, *i.e.* at a larger distance from the Ca nucleus than the previous grid point was from the B nucleus. Principally, the region around the Ca nucleus is the one where all the highest deviations are expected to be located. However, due to the larger distance of the Ca nucleus to the next grid point, the point with the shorter distance to the B nucleus displays the highest deviation at lower resolutions, until the divergence of the large deviations close to the Ca nucleus finally dominates the maximum norms at higher resolutions. This nicely illustrates the difficulties that can arise on using the maximum norm as a sole criterion of convergence behaviour.

For the smoothed distributions in the total region a uniform convergence seems to be indicated by the δ*L_n_* ^1^ and the maximum norm [Figs. 14 ▸(*a*), 14 ▸(*c*), left panels]. In contrast, the δ*L*_*n*_^2^ norm is found to diverge with increasing resolution [Fig. 14 ▸(*b*), left panel]. This contradiction may be related to the insufficient resolution of the evaluation grid. In contrast, all norm deviations in the valence region [Figs. 14 ▸(*a*)–14 ▸(*c*), right panels], after some intermediate local maximum, are found to indicate uniform convergence beyond about 2.0 Å^−1^. For the all-ED Laplacian Fourier synthesis, this is the decisive observation with respect to the chemical bonding focus of the present study.

**Figure 15:**
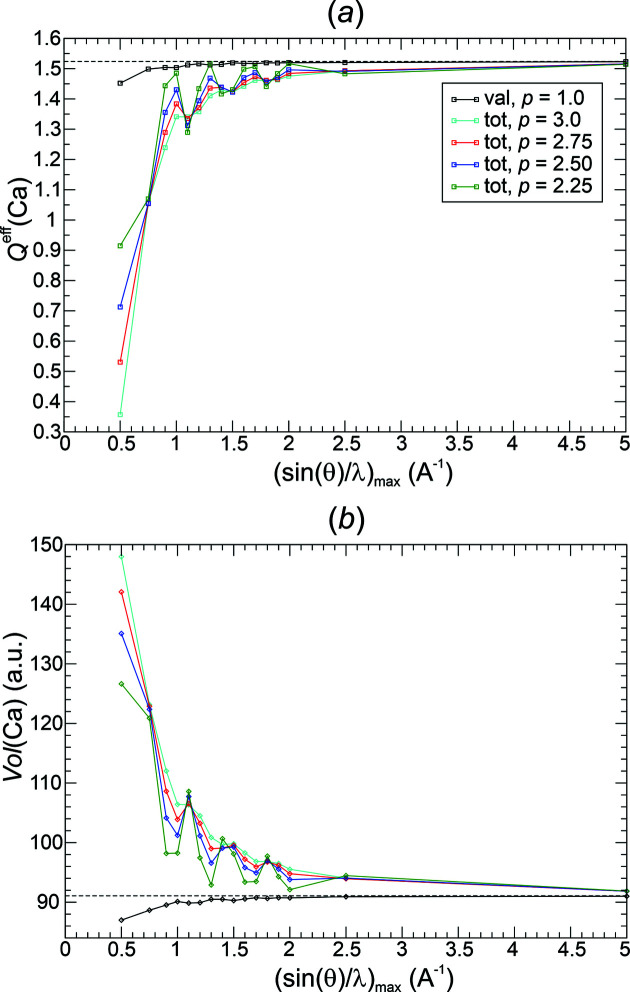
Fourier synthesis of all-ED *S_n_*_,tot_(**r**) for CaB_6_ using method Lan1D with exponents *p* = 2.25, 2.50, 2.75, 3.0 and comparison with val-ED synthesis 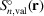 (black lines) with Lan1D(1.0); (*a*) QTAIM effective charges *Q* ^eff^(Ca), and (*b*) associated atomic volumes of the Ca atom; dashed horizontal lines indicate values obtained from the reference ED from DFT calculation.

*QTAIM basin analysis of* 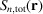 . Standard QTAIM topological analysis was performed on the all-ED distribution obtained by Fourier synthesis of the all-ED. The numerical electron recovery of the total electron number 50 e^−^/u.c. in all investigations showed an error of maximally 0.0002 electrons. The occurrence of NNMs for some resolutions (Table 2 ▸) does not lead to visible effects in the effective charge and volume curves shown (Fig. 15 ▸). The Ca effective charge obtained from QTAIM analysis of the reference DFT ED amounts to *Q* ^eff^(Ca) = +1.52.

In contrast to the rather monotonic improvement of the Ca effective charges obtained from val-ED Fourier synthesis [Fig. 15 ▸(*a*), Lan1D(1.0) val-ED results shown by the black line for comparison], all-ED synthesis features comparably large deviations improving in an oscillatory manner to the correct value [Fig. 15 ▸(*a*)]. The more aggressive variants of Lan1D (lower *p* values) Fourier synthesis are found to display the larger oscillations. They show smaller δ*L* ^1^ norm deviations [Fig. 13 ▸(*a*)] than the less aggressive ones with higher *p* values. An accuracy with respect to the Ca effective charge comparable with that found already at resolutions of about 0.75 Å^−1^ for the val-ED Fourier synthesis can be obtained with the all-ED synthesis only at resolutions beyond 2.5 Å^−1^ [Fig. 15 ▸(*a*)]. While the Ca volumes are found to be systematically too small at all resolutions for the val-ED synthesis [Fig. 7 ▸(*b*), Fig. 15 ▸(*b*) black line], the opposite is found for the all-ED synthesis [Fig. 15 ▸(*b*)]. It can be seen that the spikes of volume overestimation (*p* = 2.25) are connected with the spikes of charge underestimation (*p* = 2.25). Thus, the overestimation of the Ca atomic volumes (volume broadening) leads to the underestimation of the positive charge of Ca atomic basins (electron population enhancement).

**Figure 16:**
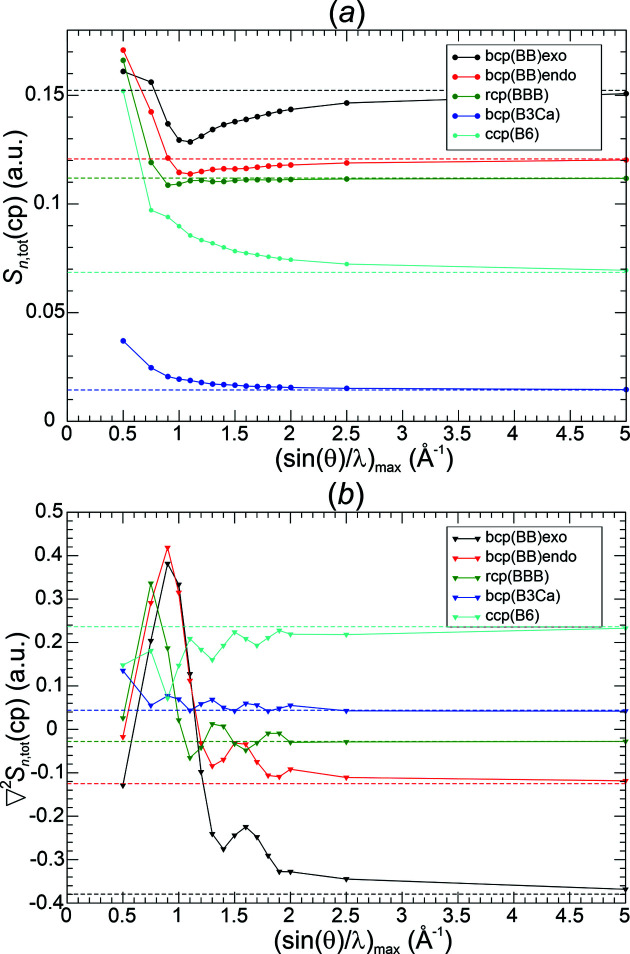
Fourier synthesis of all-ED and all-ED Laplacian of CaB_6_ using Lan1D(2.75). Critical point evaluation with respect to ED *S_n_*_,tot_(**r**_c_) (*a*) and ED Laplacian 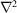 *S_n_*_,tot_(**r**_c_) (*b*) values obtained for different resolutions [sin(θ)/λ]_max_. Dashed horizontal lines indicate values obtained from the reference DFT calculation.

*Critical point analysis of* 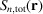 . Critical point analysis was performed for Lan1D(2.75), because for this model no NNMs adversely affect the systematic investigation. At all resolutions 0.75 Å^−1^ ≤ ½*H*_max_ ≤ 5.0 Å^−1^ shown [Fig. 16 ▸(*a*)], the reference, chemically meaningful, ED value sequence is observed for the all-ED synthesis distributions as well, Γ_8_(c.c.p.-B_6_) < Γ_6_(r.c.p.-BBB) < Γ_4_(b.c.p.-BB_endo_) < Γ_5_(b.c.p.-BB_exo_). The convergence of the ED deviations from the original values is non-monotonic for the three critical points Γ_5_, Γ_4_ and Γ_6_ with the largest ED values, displaying one local minimum at roughly ½*H*_max_ ≤ 1.1 Å^−1^, but from this point on the deviations are found to decrease monotonically with increasing resolution.

Concerning the synthesized critical point values of the all-ED Laplacian [Fig. 16 ▸(*b*)], the chemically required sequence of all-ED Laplacian values increases along Γ_5_(b.c.p.-BB_exo_) < Γ_4_(b.c.p.-BB_endo_) < Γ_6_(r.c.p.-BBB) < Γ_3_(b.c.p.-B_3_Ca) < Γ_8_(c.c.p.-B_6_), with Γ_5_, Γ_4_, Γ_6_ displaying negative values, Γ_3_ and Γ_8_ positive ones. This is observed for all resolutions ½*H*_max_ ≥ 1.7 Å^−1^.

The absolute and relative deviations of the synthesized all-ED and ED Laplacian values are given in Table 4 ▸ for resolutions ½*H*_max_ = 1.3 and 1.5 Å^−1^. Inspecting the deviations for all-ED Laplacian synthesis at ½*H*_max_ = 1.3 Å^−1^, the relative deviation of +144% at r.c.p.-BBB reveals that the chemical bonding criterion of a negative all-ED Laplacian is even missed there. This is no longer the case for resolutions ≥1.5 Å^−1^. While the absolute values of the ED deviations for Lan1D(2.75) all-ED synthesis at 1.5 Å^−1^ are of roughly similar size as those for Lan1D(2.0) val-ED synthesis at ½*H*_max_ = 1.3 Å^−1^, the corresponding all-ED Laplacian deviations for the Lan1D(2.75) all-ED synthesis are significantly higher at the three critical points, with negative reference ED Laplacian values compared with Lan1D(2.0) val-ED Laplacian synthesis. Similar to the situation discussed above for val-ED Laplacian synthesis with Lan1D(1.0), also for all-ED Laplacian synthesis using Lan1D(2.75) an oscillatory approach to the reference value is found [Fig. 16 ▸(*b*)] for critical points in the resolution range 1.0 Å^−1^ ≤ ½*H*_max_ ≤ 2.0 Å^−1^, while for resolutions beyond 2.0 Å^−1^ a rather monotonic decrease of the deviations from the reference DFT values can be expected. Also, for all-ED Laplacian synthesis, increasing the *p* value of Lan1D(2.75) is expected to smooth out this oscillatory approach at the price of increased deviations for the synthesized all-ED values. This has not been explicitly tried here. For the present study, it is considered to be sufficient to have established a rough comparison between valence- and all-ED Fourier-synthesis efficiency (Table 4 ▸) for the example of CaB_6_ and choice of synthesis method Lan1D. A wider comparison will be given in Section 4.

**Figure 17:**
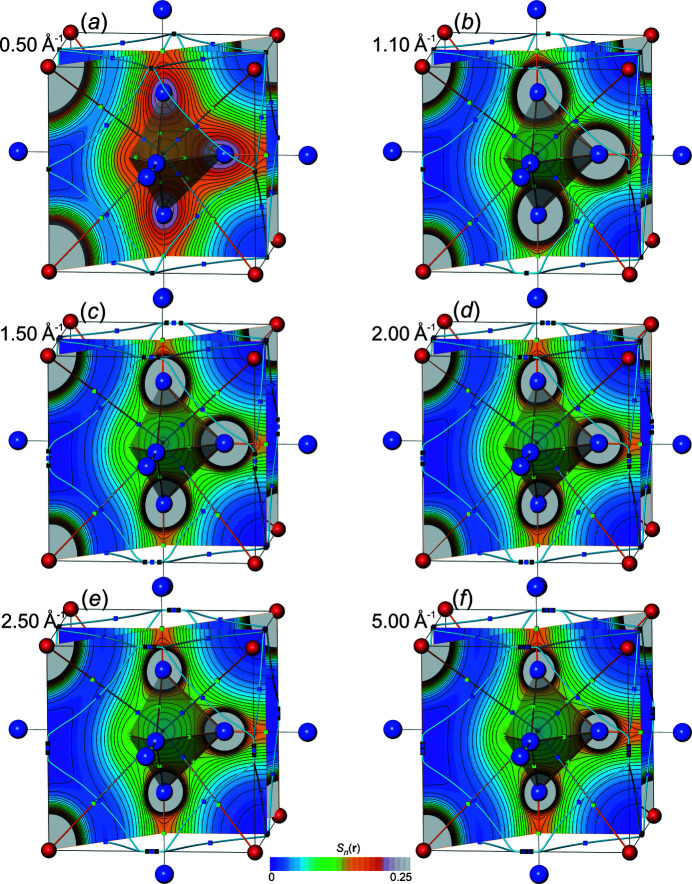
Resolution-dependent all-ED distributions 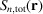 from all-ED synthesis for CaB_6_ using weighting function Lan1D(2.75). The molecular graphs with b.c.p.’s (green), r.c.p.’s (blue) and c.c.p.’s (black) are shown for each resolution; isolines are drawn at intervals of 0.01 e^−^ bohr^−3^.

*All-ED* 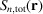 *and all-ED Laplacian* 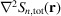 *distributions*. For visual inspection, the synthesized all-ED and all-ED Laplacian distributions are depicted in Figs. 17 ▸ and 18 ▸, respectively. It can be seen that not only the all-ED contours, but also the corresponding Laplacian contours, are very smooth. Thus, the comparably high weighting function exponent *p* = 2.75 dictated by avoidance of artefact NNMs seems to be sufficient for smoothing out Fourier-synthesis ripples as well. This is different from the val-ED synthesis situation, where the avoidance of NNMs was already achieved at *p* = 1.0, but val-ED Laplacian contour ripples at higher resolutions were clearly visible [Figs. 11 ▸(*b*), 11 ▸(*c*)]. They were seen to be smoothed out at the same exponent *p* = 2.75 as well [Figs. 11 ▸(*e*), 11 ▸(*f*)]. Nevertheless, the norm deviations for all-ED and all-ED Laplacian synthesis in the valence regions are found to be significantly higher than for val-ED [Fig. 12 ▸(*a*)] and val-ED Laplacian synthesis [Fig. 12 ▸(*b*)] using the same *exponent p* = 2.75.

## 4. Discussion

Six different weighting function methods, namely Lan3D, Pep3D, Lan1D, Fej_pcl, Fej_cnt and Fej_stl, for weighted Fourier synthesis [equations (15), (16)] have been employed for investigation of the reconstruction quality of characteristic all-ED and all-ED Laplacian features found in the corresponding reference DFT distributions of CaB_6_. Two kinds of all-ED and all-ED Laplacian reconstruction procedures have been considered: (i) Fourier synthesis of val-ED *S_n_*_,val_(**r**) and val-ED Laplacian 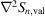 with subsequent addition [equations (19), (20)] of the corresponding reference DFT core distributions resulting in distributions 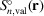 and 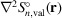 , respectively, and (ii) Fourier synthesis of all-ED *S_n_*_,tot_(**r**) and all-ED Laplacian 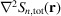 distributions.

The actual weighting functions employed are characterized by the combination of a method with a smoothing exponent *p*, denoted as *method*(*p*). It is noteworthy that, while the non-exponentiated method functions *method*(1) (*i.e.* *p* = 1) display rather different weighting curves [Fig. 4 ▸(*a*)], the combination of each method with a different exponent can make them more similar. For example, weighting function values of Fej_cnt(1.5) turned out to be very similar to the ones for Lan1D(1.0) [Fig. 4 ▸(*b*)], which was initially not the case [Fig. 4 ▸(*a*)]. Nevertheless, although overall quality, as measured by norm deviations, and chemical quality, as measured from specific QTAIM-derived deviations, were found to be very similar for both functions above, the tiny differences remaining led to non-occurrence of NNM artefacts at slightly different resolutions (Table 6 ▸).

Norm deviations of the raw and synthesized ED and ED Laplacian distributions in the whole unit-cell region have been computed to verify the mathematically expected convergence behaviours with increasing resolution. Furthermore, as an intermediate step towards the more point-wise convergence investigation at selected ED critical points in the QTAIM framework, norm deviations in the valence region of the unit cell have been studied. Considering the intended QTAIM-type topological analysis, the observed convergence of the δ*L*_*n*_^1^ norm valence-electron and all-ED deviations in the valence region [Figs. 5 ▸(*a*) and 13 ▸(*a*), right columns] is an important reconstruction quality indicator, because interatomic separatrices (basin surfaces) and critical points located on them are then embedded in a region with systematic improvement of the overall linear deviations δ*L*_*n*_^1^ (homogeneous convergence). The same is valid for the more sensible ED Laplacian distributions [Figs. 6 ▸(*a*) and 14 ▸(*a*), right columns].

Finally, the convergence of QTAIM analysis based results with increasing resolution was evaluated for each weighting function *method*(*exponent p*). The quantities considered are the effective atomic charges, and ED and ED Laplacian values at chemically important critical points. In the case of val-ED synthesis with weighting functions Lan1D(1.0), Fej_pcl(1.75), Fej_cnt(1.5) and Fej_stl(1.0) convergent behaviour is detected (Figs. 7 ▸–9 ▸). The val-ED Laplacian displays a kind of damped oscillatory approach to the reference DFT values, and the weighting functions Lan3D(1.0) and Pep3D(2.0) even showed some difficulties converging the val-ED Laplacian at selected critical points (Fig. 8 ▸) for larger resolutions (*e.g.* 5 Å^−1^), such that their occurrence in the final evaluation of reconstruction quality is only chosen for the sake of completeness, and the problematic non-converged criterion is put into brackets in Table 6 ▸. This problem of the Lan3D(1.0) scheme needs further investigation in the future.

For all-ED and all-ED Laplacian synthesis using weighting function Lan1D(2.75), convergence of the QTAIM effective charge *Q* ^eff^(Ca) was found to show oscillatory behaviour with much larger deviations than found for val-ED Lan1D(1.0) synthesis at the same resolution (Fig. 15 ▸). The reason is the large average all-ED set up by structure factor *F*_tot_(000) of 50 e^−^/*V*_u.c._, which is compared with the average val-ED of 20 e^−^/*V*_u.c._ from *F*_val_(000). This constant all-ED level must be redistributed by the subsequent structure factors to build up the comparably huge and narrow atomic ‘peak’ in the core regions and depleting the valence regions between the atoms accordingly. The redistribution process works rather slowly with increasing resolution, as has been indicated above from the synthesized values of the all-ED at the Ca position at the resolution of 5 Å^−1^ to be only 9.5% (raw synthesis) and 2% [Lan1D(2.75)] of the reference DFT value. As a consequence, the nuclear core ‘peak’ is already too wide from using raw Fourier synthesis [equations (15), (16), with constant σ^*p*^(**H** *_j_*) = 1] and even wider for smoothed Fourier synthesis [equations (15), (16), within the *method*(*exponent*) type of approach], leading to decreased charge transfer for lower resolutions, where these core-electron regions significantly overlap. For all-ED synthesis with resolutions ≥2.0 Å^−1^ *Q* ^eff^(Ca) is found to become reasonably converged (Table 6 ▸), indicating reasonably reduced core(Ca)–core(B) ED overlap. The synthesized all-ED values at the characteristic critical points investigated show rather smooth convergence behaviour for resolutions ≥1.1 Å^−1^, while all-ED Laplacian values again (like for the val-ED Laplacian) show an oscillatory approach to the reference DFT values (Fig. 16 ▸).

In a specific comparison of the reconstruction qualities of each weighting function *method*(*p*) used, a collection of four criteria has been set up; classification of the most successful function is the one fulfilling all criteria at the lowest resolution. The criteria chosen are based on chemical bonding arguments in the framework of QTAIM, and also take into account the precision necessary for typical chemical bonding discussions. They are clearly not universally objective, but serve for the predefined purpose.

The first and most important criterion for comparison of the reconstruction quality is the absence of artefact NNMs in the synthesized EDs. As already mentioned, this criterion was used to select the lowest possible smoothing exponent *p* ≥ 1.0 for each weighting *method*.

A second criterion is based on the reconstruction of the effective charges *Q* ^eff^ of the different species, *i.e.* reconstruction of *Q* ^eff^(Ca) = +1.52 obtained from QTAIM analysis of reference DFT ED is sufficient. A satisfactory recovery of the reference Ca-atom atomic charge is considered to be achieved within a deviation |Δ*Q* ^eff^(Ca)| ≤ 0.05, which is fulfilled for most val-ED Fourier-synthesis weighting functions of type *method*(*exponent*) at resolutions ≥ 0.75 Å^−1^.

Based on the reference DFT ED and chemical bonding arguments, an increase of ED at critical points in the sequence Γ_8_(c.c.p.-B_6_) < Γ_6_(r.c.p.-BBB) < Γ_4_(b.c.p.-BB_endo_) < Γ_5_(b.c.p.-BB_exo_) was required to be obeyed for a successful Fourier synthesis. This third evaluation criterion, denoted *C*{ρ(**r**_c.p._)}, is found to be obeyed at virtually all resolutions investigated (Table 6 ▸).

Satisfactory reconstruction of the val-ED Laplacian distribution is more challenging. One problem is the kind of damped oscillatory approach of the synthesized values towards the reference ones (Figs. 8 ▸, 9 ▸) found for all weighting functions [besides Pep3D(2.0)]. As investigated for method Lan1D, a suitable increase of weighting function exponent *p* damps the oscillatory approach and can thus decrease the lowest resolution for fulfilment of condition *C*{ 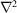 ρ(**r**_c.p._)} for the other methods with comparably small exponents 1.0 ≤ *p* < 2.0. The complete fulfilment of the requests[requirement for] of negative values of the ED Laplacian for Γ_4_, Γ_5_, Γ_6_, and positive ones for Γ_8_ and Γ_3_(b.c.p.-B_3_Ca) increasing according to Γ_5_ < Γ_4_ < Γ_6_ < Γ_3_ < Γ_8_ (which is the fourth evaluation criterion denoted *C*{ 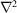 ρ(**r**_c.p._)}) is mainly dependent on the ED Laplacian sign at Γ_6_(r.c.p.-BBB). The reference value of −0.0279 bohr^−5^ is very close to zero, such that resolution-dependent, rather small variations in absolute value may yield positive ED Laplacian values here. Hence, the fulfilment of relations *C*{ 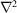 ρ(**r**_c.p._)} is strongly method and exponent dependent and is found to vary between resolutions of [sin(θ)/λ]_max_ ≥ 0.5 Å^−1^ [functions Fej_cnt(1.5) and Lan1D(1.0, 2.0)] and [sin(θ)/λ]_max_ ≥ 1.8 Å^−1^ [function Fej_pcl(1.75)].

The simultaneous fulfilment of all four conditions based on QTAIM analysis of the synthesized ED and ED Laplacian distributions of CaB_6_ leads to the conclusion that for val-ED Fourier synthesis a resolution of at least 1.2 Å^−1^ is necessary for chemically suitable ED and ED Laplacian distributions. For comparable all-ED synthesis quality, a data set resolution of 2.0 Å^−1^ is necessary (Table 6 ▸). It is noteworthy that the high value for all-ED synthesis is caused by the |Δ*Q* ^eff^(Ca)| condition and not the *C*{ 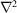 ρ(**r**_c.p._)} condition, which would have already been satisfied at and beyond 1.7 Å^−1^.

## 5. Conclusions

The topic of this pilot study is the development and evaluation of a strategy for the task of extracting sufficiently precise ED and ED Laplacian distributions using a Fourier ‘back-transformation’ process with an incomplete number of structure factors (Fourier synthesis). The compound CaB_6_, crystallizing in a cubic *cP*7-type of structure, has been chosen for this study. As a result, a Fourier-synthesis approach has been presented, which yields a systematic reconstruction of ED and ED Laplacian distributions from quantum-chemically (DFT) calculated valence-electron and all-ED static structure factors of variable resolution. The decisive issue is the application of suitable weighting functions to avoid series termination artefacts, while extracting the maximum amount (precision) of chemical bonding information possible.

The features of the ED and ED Laplacian reconstructions obtained by the present Fourier-synthesis approach have been studied for six weighting *methods*, namely Pep3D, Lan3D, Lan1D, Fej_pcl, Fej_cnt and Fej_stl. Pep3D and Lan3D have been explicitly adopted from the literature, methods Fej_pcl, Fej_cnt and Fej_stl are newly developed, and method Lan1D has been mentioned before in the literature, but not explicitly formulated. For the purpose of getting rid of NNM artefacts, a new strategy has been introduced to supplement each weighting method with a suitable smoothing factor exponent, such that the final weighting functions used were of the type *method*(*exponent*) (ME approach). The exponent *p* was adjusted to take the smallest possible value (*p* ≥ 1.0) leading to ED distributions without NNM artefacts. The smaller *p* values led to smaller norm deviations of the synthesized EDs. Using higher exponents within each method, eventual resolution-dependent ED Laplacian contour ripples can be systematically smoothed out as well, although at the price of increased ED norm deviations, such that a compromise needs to be found.

Convergence of the ED and ED Laplacian distributions with respect to the corresponding reference DFT-based distributions with increasing resolution [sin(θ)/λ]_max_ was clearly demonstrated by analysis of norm deviations of the synthesized distributions, QTAIM effective charge deviations, and ED and ED Laplacian value deviations from the reference DFT values at chemically important critical points. The criteria for successful sufficiently precise reproduction of the characteristic ED and ED Laplacian features important for chemical bonding arguments were chosen to be rather low, *e.g.* they were based on qualitative relations of values between different critical points and not on absolute value convergence. Based on these types of criteria, successful reconstruction of the val-ED and its Laplacian from valence-electron structure factors is found for resolutions ≥1.2 Å^−1^ and for all-ED structure factors ≥2.0 Å^−1^. In the evaluations of the reconstruction quality, the 1D weighting methods have overall been more successful than the 3D ones, with the best results obtained from Lan1D, Fej_pcl, Fej_cnt. This list of suitable *methods* is not considered to be a static one; it may be extended by further methods in the future, and some of the present *methods* may turn out to be less suited for, *e.g.*, systems with lower symmetry. Generally, the *methods* presented here are not suitable for a realistic reconstruction of the all-ED in the atomic core regions, because reconstruction of the steep nuclear all-ED ‘peak’ with increasing resolution is too slow. An approach complementary to the present one, focusing mainly on reproduction of the all-ED nuclear ‘peaks’, has been reported by Altomare *et al.* (2008 ▸).

The prospects of application of the presented ME type of Fourier-synthesis approach could be rather wide, provided some substantial supplementary studies are undertaken in the future.

In combination with experimental ED reconstruction studies based on the Hansen–Coppens model, the presented approach could be applied without further modification. The HC model would then play the role of the reference DFT model in the present study, and the fitted multipoles would correspond to the val-ED ρ_val_(**r**), whose static structure factors are accessible. The core-ED in the HC model is derived from free atoms and takes the role of ρ_core_(**r**) in the present study. Calculation of static ED distributions within the HC model using the multipole parameters corresponds to an extrapolation of the experimental data set to infinite resolution. Conversely, in the framework of Fourier synthesis, the application of mathematical weighting functions decaying with increasing sin(θ)/λ until [sin(θ)/λ]_max_ corresponds to systematically down-weighted contributions of the higher reflections, which could be considered as an under-interpretation of these data. Therefore, a less biased strategy for robust experimental static ED studies could be to consider both approaches in parallel. For gathering more experience with Fourier-synthesis techniques for ED and ED Laplacian reconstruction, further theoretical studies are necessary, *e.g.* for non-cubic structures.

The applicability of the presented *method*(*exponent*)-type approach to Fourier synthesis of dynamic ED and ED Laplacian distributions in the valence regions is expected. The corresponding studies could be done on the basis of the HC model using the core-electron and valence-electron structure factors and the thermal parameters. Because of the physical smoothing of the distributions caused by thermal averaging, subsequent mathematical smoothing with the present approach (eventually even with *p* ≤ 1.0) could lead to dynamical ED distributions obtained from structure-factor data sets consistent with experimental resolution avoiding data set extrapolation.

To use the presented *method*(*exponent*) type of Fourier-synthesis approach as a stand-alone technique, it also has to be complemented by a study about how to distinguish between artefact NNMs and real NNMs ‘contained’ in the structure-factor data set. For example, the degree and kind of resilience of NNMs in Fourier-synthesized (ME approach) EDs with increasing resolution should be investigated for realistic model systems with the occurrence of the NNM feature in the reference ED distributions.

## Figures and Tables

**Figure 18:**
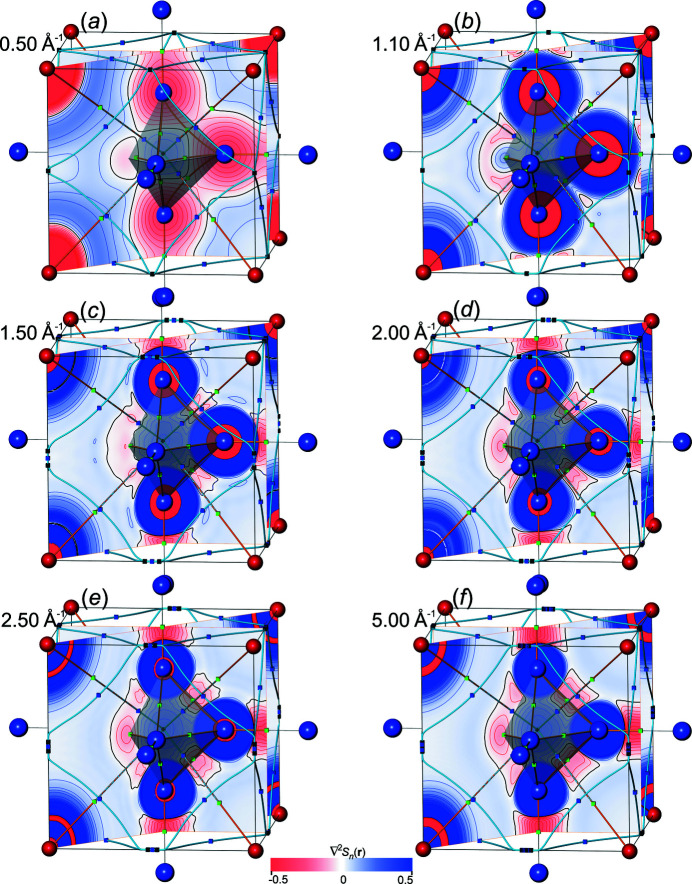
Resolution-dependent all-ED Laplacian distributions 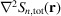 from all-ED Laplacian synthesis for CaB_6_ using weighting function Lan1D(2.75). The molecular graphs with b.c.p.’s (green), r.c.p.’s (blue) and c.c.p.’s (black) are shown for each resolution; isolines are drawn at intervals of 0.05 e^−^ bohr^−5^.

**Table 1:** Structure parameters of CaB_6_ employed for calculations (Börrnert, 2013 ▸)

Space group	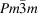 (No. 221)
Lattice parameter *a*	4.152 Å
Ca (1*a*)	0 0 0
B (6*f*)	0.2018 ½ ½

**Table 2:** *L* ^1^ and *L* ^2^ norms (in atomic units) of the reference DFT ED and ED Laplacian distributions (ρ_val_, ρ_DFT_ and 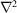 ρ_val_, 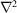 ρ_DFT_) used to calibrate the corresponding δ*L_n_* ^1^ and δ*L_n_* ^2^ norm deviations of the Fourier-synthesized distributions (*S_n_*_,val_, *S_n_*_,tot_ and 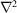 *S_n_*_,val_, 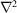 *S_n_*_,tot_)

	*m* = 1	*m* = 2
*L^m^* (**r**_u.c._, ρ_val_)	0.04140798	0.00002884
*L^m^* (**r**_u.c._, ρ_DFT_)	0.10471686	0.00204269
*L^m^* (**r**_val_, ρ_val_)	0.04533321	0.00003228
*L^m^* (**r**_val_, ρ_DFT_)	0.04700689	0.00003365
*L^m^* (**r**_u.c._, 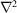 ρ_val_)	0.10144559	0.00433510
*L^m^* (**r**_u.c._, 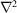 ρ_DFT_)	3.59309631	0.23552473
*L^m^* (**r**_val_, 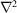 ρ_val_)	0.05329431	0.00004829
*L^m^* (**r**_val_, 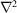 ρ_DFT_)	0.08058963	0.00014996

**Table 3:** Fourier synthesis of val-ED *S_n_*_,val_(**r**) for CaB_6_ Location of NNMs (see column ‘location’) and QTAIM basin populations from 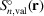 for all methods (exponents *p*) and resolutions ½*H*max used.

Method	½*H*_max_ (Å^−1^)	Population (e^−^)	Location
Lan3D, *p* = 1.0	5.0	24/4 × 0.0000	Split Ca—Ca
	1.0	6/2 × 0.6255	B—B_exo_
	0.9	6 2 × 0.6455	B—B_exo_
	0.75	6 2 × 0.7170	B—B_exo_
Lan1D, *p* = 1.0	1.0	6/2 × 0.6951	B—B_exo_
	0.9	6/2 × 0.6961	B—B_exo_
	0.75	6/2 × 0.6902	B—B_exo_
*p* = 2.0	1.2	6/2 × 0.6219	B—B_exo_
	1.1	6/2 × 0.6509	B—B_exo_
	1.0	6/2 × 0.6435	B—B_exo_
	0.9	6/2 × 0.5901	B—B_exo_
	0.75	6/2 × 0.4243	B—B_exo_
	0.5	6 × 0.6057	Split B_6_^oct^ midpoint
* p* = 2.75	1.1	6/2 × 0.5941	B—B_exo_
	0.5	1 × 4.4717	B_6_^oct^ midpoint
Fej_pcl, *p* = 1.75	1.1	6 × 0.3479	Split B—B_exo_
	1.0	6/2 × 0.7589	B—B_exo_
	0.9	6/2 × 0.7790	B—B_exo_
	0.75	6/2 × 0.8307	B—B_exo_
	0.5	6/2 × 0.0606	B—B_exo_
Fej_cnt, *p* = 1.50	1.1	6 × 0.2783	Split B—B_exo_
	1.0	6/2 × 0.7004	B—B_exo_
	0.9	6/2 × 0.6957	B—B_exo_
	0.75	6/2 × 0.6945	B—B_exo_
Fej_stl, *p* = 1.0	1.6	6/2 × 0.1777	B—B_exo_
	1.0	6/2 × 0.5437	B—B_exo_
	0.9	6/2 × 0.5608	B—B_exo_
	0.75	6/2 × 0.5785	B—B_exo_
Pep3D, *p* = 2.0	5.0	1 × 0.0012	B_6_^oct^ midpoint
	0.5	1 × 4.3702	B_6_^oct^ midpoint

**Table 4:** Deviations of Lan1D(*p*) synthesized val-EDs and all-EDs and their Laplacians from reference DFT values at chemically important critical points for selected resolutions and exponents *p* Values given in the first row correspond to the reference DFT values ρ(**r**_c.p._) and 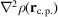 at each critical point; values given in each cell of the table body correspond to absolute and relative deviations [equations (44), (45)] of the ED (top row) and the ED Laplacian (bottom row), respectively; ED values and differences given in e^−^ bohr^−3^, ED Laplacian ones in e^−^ bohr^−5^.

ρ_DFT_(**r**_c.p._), 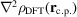	b.c.p.-BB_exo_ 0.1523, −0.3794	b.c.p.-BB_endo_ 0.1207, −0.1250	r.c.p.-BBB 0.1119, −0.0279	c.c.p.-B_6_ 0.0685, +0.2363	b.c.p.-B_3_Ca 0.0144, +0.0439
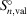 , 1.1 Å^−1^	−0.0107, −7.1%	−0.0035, −2.9%	−0.011, −1.0%	0.0074, 10.9%	0.0005, 3.3%
*p* = 1.0	0.0487, 12.8%	0.0290, 23.2%	0.0245, 87.7%	−0.0460, −19.5%	0.0014, 3.2%
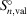 , 1.3 Å^−1^	−0.0153, −10.1%	−0.0049, −4.1%	−0.0012, −1.1%	0.0098, 14.3%	0.0006, 4.2%
*p* = 2.0	0.0739, 19.5%	0.0319, 25.5%	0.0018, 6.5%	−0.0343, −14.5%	0.0034, 7.7%
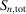 , 1.3 Å^−1^	−0.0180, −11.9%	−0.0048, −4.0%	−0.0016, −1.4%	0.0134, 19.6%	0.0027, 18.9%
*p* = 2.75	0.1387, 36.6%	0.0405, 32.4%	0.0403, 144%	0.0763, 32.3%	0.0245, 55.9%
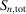 , 1.5 Å^−1^	−0.0144, −9.5%	−0.0046, −3.8%	−0.0011, −1.0%	0.0098, 14.3%	0.0022, 15.4%
*p* = 2.75	0.1355, 35.7%	0.0936, 74.9%	−0.0051, 18.4%	−0.0124, −5.3%	−0.0014, −3.1%

**Table 5:** Fourier synthesis of all-ED Location of NNMs and their basin populations for Lan1D method using various exponents 2.25 ≤ *p* ≤ 3.0.

Method(*p*)	½*H*_max_ (Å^−1^)	Population (e^−^)	Location
Lan1D, *p* = 2.25	1.9	6/2 × 0.1446	B—B_exo_
	1.8	6/2 × 0.1655	B—B_exo_
	1.3	6/2 × 0.2559	B—B_exo_
	1.2	6/2 × 0.2850	B—B_exo_
		12 × 0.1624	B—B_endo_
Lan1D, *p* = 2.50	1.3	6/2 × 0.1239	B—B_exo_
	1.1	8 × 0.0141	B_3_^triangle^
Lan1D, *p* = 2.75	–	–	–
Lan1D, *p* = 3.00	–	–	–

**Table 6:** Summary of numerical quality criteria fulfilment based on QTAIM analysis for various weighting functions *method*(*exponent p*) tested Values are given for val-ED synthesis (‘*S_n_*_,val_ synthesis’) and all-ED synthesis (‘*S_n_*_,tot_ synthesis’). Resolutions for criterion *C*{ 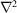 ρ(**r**_c.p._)} are put into brackets, where the ED Laplacian at critical points does not clearly converge. For each *method*(*exponent*) the decisive resolution for fulfilment of all four criteria is marked in bold.

	Resolutions free from NNM (Å^−1^)	Resolutions with |Δ*Q* ^eff^(Ca)| ≤ 0.05 (Å^−1^)	Resolutions obeying *C*{ρ(**r**_c.p._)} (Å^−1^)	Resolutions obeying *C*{ 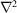 ρ(**r**_c.p._)} (Å^−1^)
*S_n_*_,val_ synthesis:				
Lan3D, *p* = 1.0	≥ 1.1	≥ 0.75	≥ 0.75	(≥ **1.3**)
Lan1D, *p* = 1.0	≥ **1.1 **	≥ 0.75	≥ 0.75	≥ 0.5
Lan1D, *p* = 2.0	≥ **1.3**	≥ 0.75	≥ 0.75	≥ 0.5
Fej_pcl, *p* = 1.75	≥ 1.2	≥ 0.75	≥ 0.5	≥ **1.8**
Fej_cnt, *p* = 1.5	≥ **1.2**	≥ 0.75	≥ 0.75	≥ 0.5
Fej_stl, *p* = 1.0	≥ 1.7	> **5.0**	≥ 0.75	≥ 1.7
Pep3D, *p* = 2.0	≠ 0.5, **5.0**	≥ **5.0**	≥ 1.3	(≥ 0.75)
*S_n_*_,tot_ synthesis:				
Lan1D, *p* = 2.75	≥ 0.5	≥ **2.0**	≥ 0.75	≥ 1.7

## Appendix A Electron-density and structure-factor calculation from wavefunctions

Periodic DFT calculations of the electronic structure of CaB_6_ were carried out using the experimentally determined lattice parameter of 4.152 Å and a boron position at 6*f* (*x*, ½, ½) with *x* = 0.2018 (Börrnert, 2013 ▸). Wavefunctions were calculated at the DFT/PBE (Perdew *et al.*, 1996 ▸) level based on the full-potential augmented plane wave (APW) method with local orbitals using the full potential (L)APW code *Elk* (Dewhurst *et al.*, 2011 ▸). A (4, 4, 4) *k*-point set was used. For calcium, an APW+lo-basis was used, where atomic 1*s*, 2*s*, 2*p* states were technically treated as core states. For boron atoms, a LAPW+lo+LO basis was used, where all electrons were technically treated as valence electrons. The ED of the inner part of the MT (muffin-tin) spheres was expanded with a maximal angular momentum lVmax = 6 (Baranov & Kohout, 2011 ▸). The interpolation step width for the radial points within the spheres was decreased to 1. The maximum length of the **G**+**k** vector divided by the smallest MT radius (RGkAPWmax) was 10.0, the angular momentum cut-off for the MT electron density and potential (lVmax) was 12.0, the maximal length of the G-vector for the expansion of the interstitial electron density and potential (GVmax) was 26.0, and the angular momentum cut-off for the APW functions (lAPWmax) was 12.0.

The DOS (density of states) of CaB_6_ reveals a clear energetic separation of 9 eV for chemical valence states (20 electrons per formula unit) and core states [B(1*s* ^2^), Ca(1*s* ^2^, 2*s* ^2^, 2*p* ^6^, 3*s* ^2^, 3*p* ^6^)] (Börrnert, 2013 ▸). Therefore, besides the all-electron structure factors, the chemical valence-electron structure factors of CaB_6_ were calculated, applying a suitable energy window for their calculation with *Elk*.

The ED and its Laplacian calculated from the DFT wavefunction were obtained with the *DGrid-4.7* code (Kohout, 2012 ▸). In addition, a module performing the weighted Fourier synthesis according to equations (7)–(16) has been implemented into *DGrid-4.7* (Börrnert *et al.*, 2022 ▸). With that module, real-space distributions of ED and the ED Laplacian and subsequent QTAIM analysis were available from the reference DFT wavefunction (reference DFT distributions and values) as well as from the structure-factor sets of varying resolution and with varying weighting functions *method*(*exponent*) (Fourier-synthesized distributions and values). While the QTAIM basin boundaries are given with the grid resolution used (uniform mesh with mesh size of 0.02628 Å), the ED integration inside these boundaries was obtained at much better spatial resolution by invoking the standard ED refinement algorithm of *DGrid-4.7*.
